# Resistance to the Tat Inhibitor Didehydro-Cortistatin A Is Mediated by Heightened Basal HIV-1 Transcription

**DOI:** 10.1128/mBio.01750-18

**Published:** 2019-07-02

**Authors:** Guillaume Mousseau, Rachna Aneja, Mark A. Clementz, Sonia Mediouni, Noemia S. Lima, Alexander Haregot, Cari F. Kessing, Joseph A. Jablonski, Suzie Thenin-Houssier, Nisha Nagarsheth, Lydie Trautmann, Susana T. Valente

**Affiliations:** a Department of Immunology and Microbiology, The Scripps Research Institute, Jupiter, Florida, USA; b Henry M. Jackson Foundation for the Advancement of Military Medicine, Bethesda, Maryland, USA; c U.S. Military HIV Research Program, Walter Reed Army Institute of Research, Silver Spring, Maryland, USA; Gladstone Institutes; Albert Einstein College of Medicine

**Keywords:** HIV promoter, HIV transcription, Nef, Tat inhibitor, Vpr, drug resistance, viral resistance

## Abstract

Human immunodeficiency virus type 1 (HIV-1) Tat binds the viral RNA structure transactivation-responsive element (TAR) and recruits transcriptional cofactors, amplifying viral mRNA expression. The Tat inhibitor didehydro-cortistatin A (dCA) promotes a state of persistent latency, refractory to viral reactivation. Here we investigated mechanisms of HIV-1 resistance to dCA *in vitro*. Mutations in Tat and TAR were not identified, consistent with the high level of conservation of these elements. Instead, viruses resistant to dCA developed higher Tat-independent basal transcription. We identified a combination of mutations in the HIV-1 promoter that increased basal transcriptional activity and modifications in viral Nef and Vpr proteins that increased NF-κB activity. Importantly, these variants are unlikely to enter latency due to accrued transcriptional fitness and loss of sensitivity to Tat feedback loop regulation. Furthermore, cells infected with these variants become more susceptible to cytopathic effects and immune-mediated clearance. This is the first report of viral escape to a Tat inhibitor resulting in heightened Tat-independent activity, all while maintaining wild-type Tat and TAR.

## Abstract (precis)

HIV-1 Tat enhances viral RNA transcription by binding to TAR and recruiting activating factors. Tat enhances its own transcription via a positive-feedback loop. Didehydro-cortistatin A (dCA) is a potent Tat inhibitor, reducing HIV-1 transcription and preventing viral rebound. dCA activity demonstrates the potential of the “block-and-lock” functional cure approaches. We investigated the viral genetic barrier to dCA resistance *in vitro*. While mutations in Tat and TAR were not identified, mutations in the promoter and in the Nef and Vpr proteins promoted high Tat-independent activity. Promoter mutations increased the basal transcription, while Nef and Vpr mutations increased NF-κB nuclear translocation. This heightened transcriptional activity renders CD4^+^ T cells infected with these viruses more susceptible to cytotoxic T cell-mediated killing and to cell death by cytopathic effects. Results provide insights on drug resistance to a novel class of antiretrovirals and reveal novel aspects of viral transcriptional regulation.

## INTRODUCTION

Human immunodeficiency virus type 1 (HIV-1) has the remarkable ability to escape selective pressure (1–3); as such, it is very common for HIV to become resistant to all classes of antiretrovirals (ARVs) when used individually (4–7). To prevent resistance development, antiretroviral therapy (ART) is used as a triple or quadruple combination of ARVs (8, 9). Despite ART’s ability to reduce HIV to very low levels, it cannot eradicate HIV. The virus persists in latently infected memory CD4^+^ T cells, even in individuals with undetectable plasma viremia (10, 11). Currently, no ARV inhibits transcription of the integrated provirus and reactivation from latency upon treatment interruption, highlighting the need to develop this class of drugs.

Basal transcription from the integrated HIV long terminal repeat (LTR) is modest; however, the viral transactivator of transcription (Tat) and host factors exponentially increase viral mRNA production (12–17). We discovered didehydro-cortistatin A (dCA), the equipotent analogue of the natural product cortistatin A (CA), as a potent inhibitor of Tat (half-maximal inhibitory response [IC_50_] = 1 nM) (18). Both dCA and CA selectively inhibit Tat-mediated transactivation of the integrated HIV provirus (18, 19). Over time, dCA treatment drives viral gene expression into an induced state of persistent latency *in vitro,* as epigenetic modifications progressively accrue at the HIV-1 promoter (20). dCA renders primary latently infected CD4^+^ T cells, isolated from individuals on suppressive ART, refractory to viral reactivation by a panel of latency reversing agents (21). We have shown that adding dCA to an ART-suppressed humanized mouse model of HIV-1 latency systemically reduces viral RNA in tissues and significantly delays and reduces viral rebound levels upon treatment interruption (22). These findings support the “block-and-lock” functional cure for HIV-1. Specifically, transcriptional inhibitors could promote a state of sustained latency, halting ongoing viral transcription during ART and blocking reactivation of the latent provirus (23, 24).

Here we investigated the genetic barrier to HIV-1 strain NL4-3 resistance to dCA *in vitro*. We identified two isolates presenting mutations in the LTR promoter that increased basal transcriptional activity and nucleotide changes in Nef and Vpr that increased NF-κB activity. These variants are unlikely to enter latency, and their high transcription levels renders them more cytopathic and more susceptible to cytotoxic T cell (CTL)-mediated immune clearance *in vitro* and possibly *in vivo*. To our knowledge, the present study describes the first HIV-1 isolate resistant to a Tat inhibitor, in which Tat and transactivation-responsive element (TAR) remain intact, and provides important insights into possible alternative mechanisms that may regulate HIV-1 transcription.

## RESULTS

### Generation of viral isolates resistant to the Tat inhibitor dCA.

**FIG 1:**
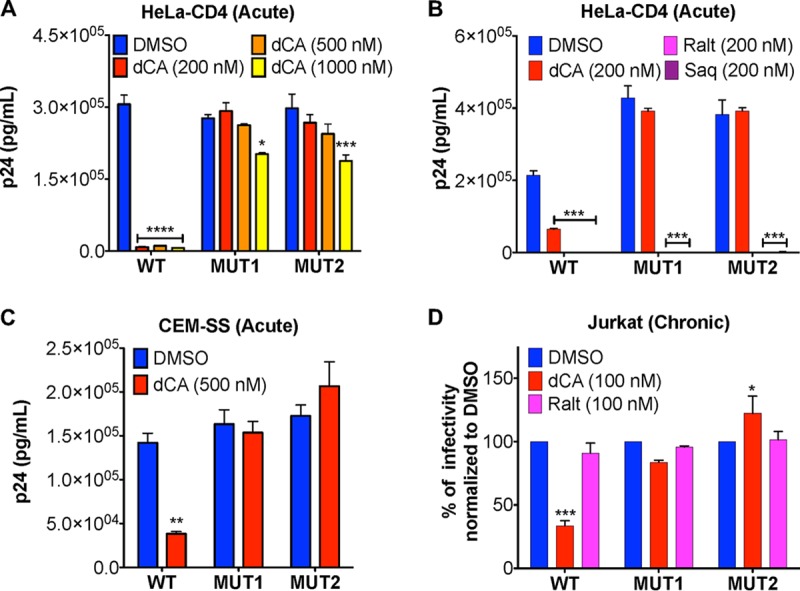
HIV-1 isolates MUT1 and MUT2 are resistant to the Tat inhibitor dCA. (A) Inhibition of WT NL4-3 but not of the natural isolates MUT1 and MUT2 by increasing concentrations of dCA. HeLa-CD4 cells were infected with viruses, washed, and treated with the indicated concentrations of dCA or DMSO control. Supernatants collected 48 h later to quantify p24 capsid by ELISA (*n* = 3). (B) The dCA-resistant natural isolates MUT1 and MUT2 remain sensitive to integrase and protease inhibitors. Supernatants were collected 96 h later to quantify p24 capsid by ELISA (*n* = 3). (C) Viral resistance to dCA is cell type independent. CEM-SS cells were infected with the indicated viruses and then handled as described above for panel A. Supernatants harvested 72 h later were subjected to p24 ELISA (*n* = 3). (D) Chronically infected Jurkat cells were treated with the indicated compounds for 4 days before the levels of p24 in the supernatants were assessed by ELISA (*n* = 4). Data are normalized to the values for 100% DMSO control for each virus. All data are presented as means plus standard errors of the means (SEM). The two-way ANOVA followed by a Bonferroni posttest were used for statistical comparisons. Values that are significantly different are indicated by asterisks as follows: *, *P* < 0.05; **, *P* < 0.01; ***, *P* < 0.001; ****, *P* < 0.0001. Abbreviations: WT, wild type; MUT, mutant (dCA-resistant natural isolate); Ralt, raltegravir; Saq, saquinavir.

Viruses resistant to dCA (EC_50_ = 1 nM) were obtained by passaging the viral isolate NL4-3, every week, onto naive HeLa-CD4 cells, in the presence of increasing concentrations of dCA (from 0.1 nM to 1 μM), over a 12-month period. Resistance to dCA developed slowly for approximately 9 months, when cultured between 0.1 and 1 nM concentrations of dCA, and faster during the next 3 months, when using 10 nM to 1 μM concentrations of dCA. Two independent virus populations were isolated and named MUT1 and MUT2 (MUT for mutant). These viruses replicated efficiently in 1 μM dCA, compared to the sensitive wild-type (WT) NL4-3 control (Fig. 1A). As expected, the two dCA-resistant viral populations retained sensitivity to both the integrase inhibitor raltegravir and the protease inhibitor saquinavir (Fig. 1B). MUT1 and MUT2 also replicated efficiently in the presence of 500 nM concentration of the natural compound, CA (see Fig. S1 in the supplemental material). To exclude a cell-type-dependent event, resistant isolates were also tested onto the T-lymphoblastic cell line CEM-SS (Fig. 1C). Upon acute infection, both MUT1 and MUT2 replicated equally well in CEM-SS cells, in the presence of DMSO or 500 nM dCA. A Tat inhibitor’s key feature is its unique ability to inhibit transcription from infected cells with stable proviral integration termed “chronic” (18, 21). As such, while WT viral transcription was inhibited from chronically infected Jurkat cells treated with 100 nM dCA, chronic infections with MUT1 and MUT2 were unaffected (Fig. 1D). Raltegravir was used as a negative control to ensure the chronic state of these cells. Altogether, our results suggest that the transcription from both MUT1 and MUT2 is unaffected by the presence of up to 1,000-fold the EC_50_ concentration of dCA, in a cell-type-independent manner.

10.1128/mBio.01750-18.1 FIG S1 The natural isolates MUT1 and MUT2 are resistant to the natural product cortistatin A (CA). HeLa-CD4 cells were infected with WT, MUT1, or MUT2 virus and treated with DMSO or 500 nM CA. Supernatants were harvested 48 h later, and viral capsid in the supernatant was quantified by p24 ELISA (*n* = 3). All data are presented as mean ± SEM. The two-way ANOVA followed by a Bonferroni posttest were used for statistical comparisons. *, *P* < 0.05; **, *P* < 0.01; ***, *P* < 0.001. Download FIG S1, PDF file, 0.04 MB. Copyright © 2019 Mousseau et al. 2019 Mousseau et al. This content is distributed under the terms of the Creative Commons Attribution 4.0 International license.

### Identification of mutations conferring resistance to dCA.

**FIG 2:**
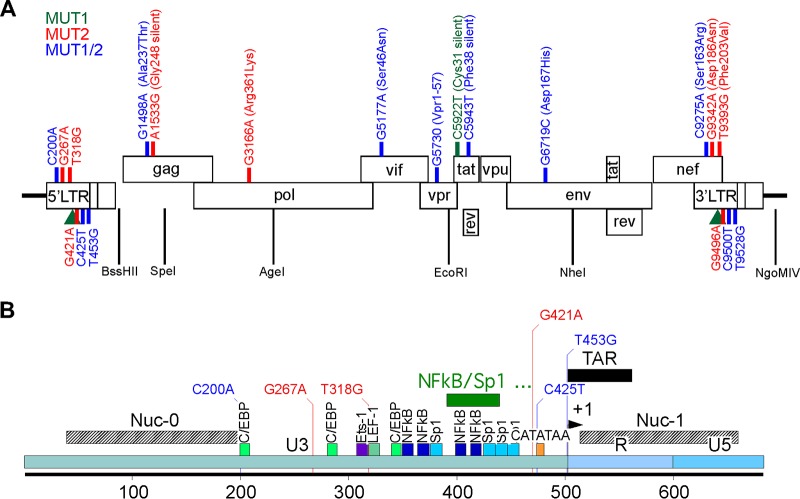
Schematic representation showing the positions of the mutated nucleotides in MUT1 and MUT2 dCA-resistant HIV-1 isolates. (A) NL4-3 genome with the indicated mutations found after deep sequencing, representing 50% or higher frequency of nucleotide changes. Green, red, and blue lines indicate the positions of mutations in MUT1, MUT2 and both MUT1/MUT2, respectively. The green triangle is a two NF-κB/one Sp1 insertion detected in MUT1. Amino acid changes are also indicated where applicable. The restriction sites used for cloning are indicated. (B) Close-up of the NL4-3 5′ LTR, using the same color code as in panel A.

The WT, MUT1, and MUT2 genomes were isolated from viral particles and sequenced using Next Generation Sequencing Illumina technology, generating an average 5,000× coverage of the 10-kb HIV genome. A consensus sequence that represents 50% or higher frequency of nucleotide change revealed 9 point mutations and 2 NF-κB/one Sp1 insertion in the LTR in MUT1 and 13 mutations in MUT2 (Fig. 2). Mutations were identified in the LTR region as well as in Gag, Pol, Vif, Vpr, Tat, Env and Nef genes (Fig. 2 and Table 1). At the protein level, mutations in Gag included Ala237Thr, at the N terminus of the p24 capsid, and one silent mutation. The Pol gene in MUT2 included one synonymous change (Arg361Lys) in the reverse transcriptase basic loop of the RNase H domain. One mutation was observed in Vif (Ser46Asn), and the nucleotide change G5177A in the Vpr gene resulted in a truncation (Vpr_1-57_), which removes the third helix and the flexible C-terminal portion of the protein. Mutations in both MUT1 and MUT2 Tat protein were silent. In the gp120, one amino acid change (Asp167His) in the V2 loop was identified. Finally, three mutations in the LTR region overlap with changes in the Nef protein (Ser163Arg, Asp186Asn and Phe203Val).

**TABLE 1:** MUT1 and MUT2 mutations^*a*^

Location	Nucleotide mutation(s)	Amino acid change	Found in	Comment(s)
5′LTR/3′LTR/Nef	C200A/C9275A	Ser163Arg	MUT1/MUT2	Nucleotide located at the 3′ extremity of the 5′ LTR nucleosome 0 binding site (67, 68).
				Corresponds to the first nucleotide of a putative 5′ LTR C/EBP binding site (69, 70).
				Residues in the Nef dileucine motif ENTSLL known to interact with AP-2 protein and downregulate CD4 (55).
	G267A/G9342A	Asp186Asn	MUT2	Mutation of a main Nef residue in the β-catenin binding motif D186-X-X-X-X-[F/Y191] (59).
	T318G/T9393G	Phe203Val	MUT2	Mutation of the first nucleotide of the consensus TCF-4/LEF-1 binding site TACAAAG, site of the TCF-4/β-catenin/SMAR1 complex known to repress HIV-1 transcription (71, 72).
				Tyrosine motif Tyr202/[Phe/Tyr]203 in the C-terminal flexible loop of Nef is involved in CD4 receptor endocytosis (61).
5′LTR/3′LTR	49-nt insertion^*b*^ downstream of nt 390 and 9465		MUT1	Two NF-κB/one Sp1 insertion.
	G421A/G9496A		MUT2	Upstream (position −4) of the TATA box.
	C425T/C9500T		MUT1/MUT2	Mutation of the HIV-1 TATA box CATATAA at position +1 to TATATAA known to slightly increase viral fitness (39).
	T453G/T9598G		MUT1/MUT2	Position −2 of the transcription start site (TSS) and at the basis of the TAR hairpin.
Gag	G1498A	Ala237Thr	MUT1/MUT2	Found in the N-terminal domain of mature p24 capsid protein (73).
	A1533G	Gly248 (silent)	MUT2	
Pol	G3166A	Arg361Lys	MUT2	Found in the basic loop of the RNase H domain of the reverse transcriptase (74).
Vif	G5177A	Ser46Asn	MUT1/MUT2	Residue part of the ^45^ESTN^48^ motif shown to play a role in CBF β interaction (75).
Vpr	G5730 deletion	C-terminal truncation resulting in Vpr_1-57_ plus LysPro in the C terminus	MUT1/MUT2	The Vpr truncation (aa 58 to 96) encompasses the third helix of Vpr (aa 56 to 77) and its flexible C-terminal region (76).
Tat	C5922T	Cys31 (silent)	MUT1	
	C5943T	Phe48 (silent)	MUT1/MUT2	
Env	G6719C	Asp167His	MUT1/MUT2	Residue in the V2 loop of Env gp120. Epitope for neutralizing antibodies (77).

a Numbering is based on the pNL4-3 sequence (GenBank accession no. AF324493.2). Amino acid numbering is based on each individual viral protein. nt, nucleotides; aa, amino acids.

b The 49 nucleotides inserted are TGCTACAAGG AACTTTCCGC TGGGGACTTT CCAGGGAGGC GTGGCCTGG.

### Resistant viruses have higher replication fitness resulting in higher cytotoxicity.

**FIG 3:**
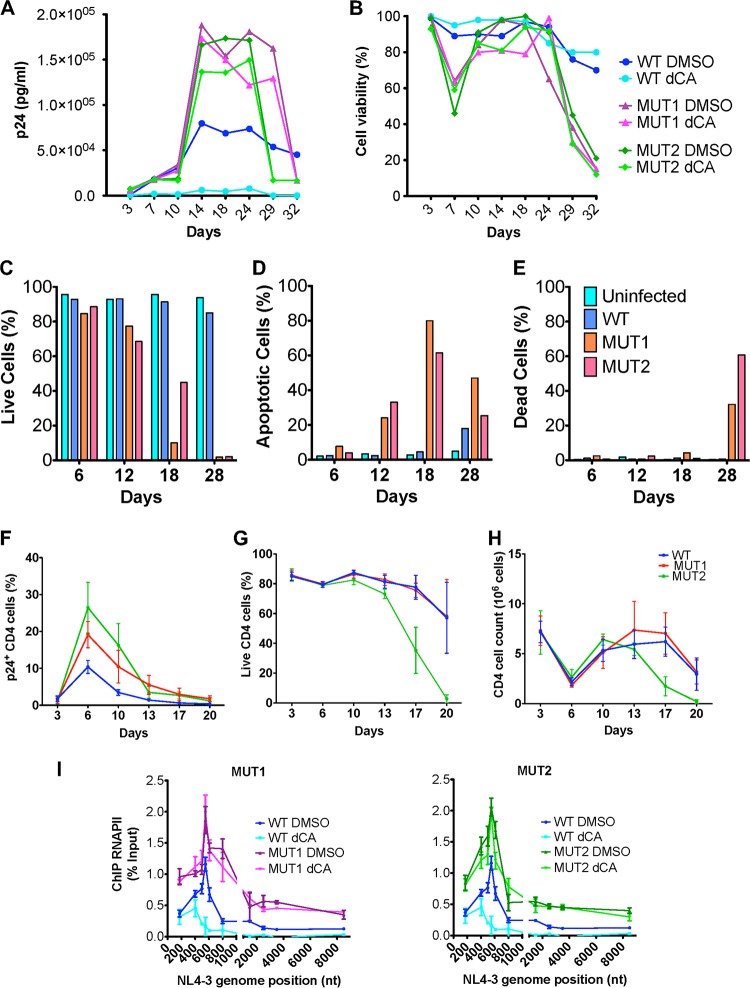
Cells infected with dCA-resistant viruses have high replication rates and eventually undergo cytopathic cell death. (A and B) HeLa-CD4 cells were infected with WT or dCA-resistant MUT1 and MUT2 viruses for 8 h. Cells were then treated with DMSO or 100 nM dCA. (A) Viral capsid quantified by p24 ELISA and (B) viability of infected HeLa-CD4 cells monitored using trypan blue cell staining. The results shown are representative of three independent experiments. (C to E) Analysis by flow cytometry of the cell viability of acutely infected HeLa-CD4 cells. HeLa-CD4 cells uninfected and infected with WT, MUT1 and MUT2 viruses were stained with annexin V and Zombie Red. Zombie Red-negative annexin V-negative cells represent viable cells. Flow cytometry analysis was performed over time. Data are representative of three independent experiments. (F to H) Primary CD4^+^ T cells from three independent donors were infected with WT or dCA-resistant MUT1 and MUT2 viruses. At different times postinfection, cells were stained and analyzed by flow cytometry. (F) Frequency of CD4^+^ T cells expressing p24; (G) frequency of Live/Dead-negative CD4^+^ T cells assessed by flow cytometry; and (H) absolute number of CD4^+^ T cells in culture assessed by cell counting with trypan blue staining. Genome positions are shown in nucleotides (nt). (I) RNAPII recruitment to dCA-resistant virus isolates assessed by ChIP. HeLa-CD4 cells were infected with WT, MUT1 or MUT2 viruses in the presence of DMSO or 100 nM dCA, and 9 days postinfection, the cells were cross-linked and ChIP to RNAPII was performed. The promoter of RPL13A was used as a reference (see Fig. S3 in the supplemental material). The results are presented as percent immunoprecipitated DNA over input. All data are presented as means ± SEM (*n* = 3).

To investigate the replication rate of the viruses, we infected HeLa-CD4 cells with WT, MUT1 or MUT2 viruses and monitored their viral replication over time (Fig. 3A). Starting at the same multiplicity of infection as used for the WT, both MUT1 and MUT2 replicated at a higher rate (two- to three-fold) than the WT control. This high replication rate eventually lead to drastic cytopathic cell death (Fig. 3B), while the WT control eventually maintained a chronic infection. As expected, dCA inhibited only the WT infection. To further characterize the cytopathic effect, in an independent experiment, the signals of apoptosis and cell death were measured by flow cytometry at different time points postinfection (Fig. 3C to E). Apoptosis was monitored by annexin V staining, known to bind to the phosphatidylserines externalized at the membranes of apoptotic cells (Fig. 3D). The viability of infected HeLa-CD4 cells was assessed using a Zombie Red dye only permeable to dead cells (Fig. 3E). The number of live cells decreased considerably over time upon MUT1 and MUT2 infections, with a peak of apoptosis at day 18 and total cell loss by day 28 (Fig. 3C to E). Specific flow cytometry panels showing gating and time course are shown in Fig. S2.

10.1128/mBio.01750-18.2 FIG S2 Evaluation of cell viability by flow cytometry. HeLa-CD4 cells acutely infected with WT, MUT1, and MUT2 viruses were passaged every 3 days. Hela-CD4 cells were stained with a cocktail of two antibodies, namely, APC-labeled antibody to annexin V and Live/Dead staining Zombie Red at days 6, 12, 18, and 28. HeLa-CD4 cells were gated by forward and side scatter. Live, apoptotic and dead cells were resolved in the FL2 (Zombie Red) and FL4 (annexin V) flow channels (*n* = 1). Download FIG S2, PDF file, 0.3 MB. Copyright © 2019 Mousseau et al. 2019 Mousseau et al. This content is distributed under the terms of the Creative Commons Attribution 4.0 International license.

10.1128/mBio.01750-18.3 FIG S3 Recruitment of RNAPII to RPLI3A promoter in acutely infected HeLa-CD4 cells. ChIP to RNAPII was performed 9 days postinfection of Hela-CD4 cells infected with WT, MUT1, or MUT2 virus in the presence of DMSO or 100 nM dCA. Recruitment of RNAPII to ribosomal gene RPLI3A was used as a control (Fig. 3). All data are presented as mean ± SEM (*n* = 2). Download FIG S3, PDF file, 0.04 MB. Copyright © 2019 Mousseau et al. 2019 Mousseau et al. This content is distributed under the terms of the Creative Commons Attribution 4.0 International license.

Next, we investigated this cytopathic effect in primary CD4^+^ T cells isolated from three independent individuals. Primary CD4^+^ T cells infected with MUT1 and MUT2 viruses showed higher frequencies of cells expressing p24 capsid than cells infected with WT virus (Fig. 3F). Results represent the averages of the cells from three individuals. Cell viability decreased faster in CD4^+^ T cells infected with MUT2 than those infected with WT virus (Fig. 3G and H). Interestingly, in primary cells, overt cytotoxicity, at the multiplicity of infection used for MUT1, was not readily observed, which also correlated with lower intracellular p24 expression.

To investigate the relation between increased replication rate of dCA-resistant viruses and HIV-1 transcription, we assessed the recruitment of the RNA polymerase II (RNAPII) to the HIV promoter and open reading frame (ORF) by chromatin immunoprecipitation (ChIP). HeLa-CD4 cells were infected with WT, MUT1, and MUT2 viruses, and 9 days after infection, cells were analyzed by ChIP. In the absence of dCA, RNAPII recruitment to the promoter and ORF of MUT1 and MUT2 resistant viruses was increased by 1.5- to 2.0-fold compared to WT virus (Fig. 3I). In the presence of dCA, RNAPII recruitment to the HIV-1 promoter and the ORF of WT virus was drastically inhibited, while dCA had no impact on RNAPII recruitment to the MUT1 and MUT2 genome. For a control, no detectable variation of RNAPII recruitment at the promoter of the housekeeping gene RPL13A was observed (Fig. S3). Collectively, these results suggest that dCA-resistant viruses, with a heightened transcriptional fitness, are more cytopathogenic and may have lost the ability to regulate transcription and enter into latency.

### dCA-resistant viruses render infected CD4^+^ T cells more susceptible to CTL-mediated killing.

**FIG 4:**
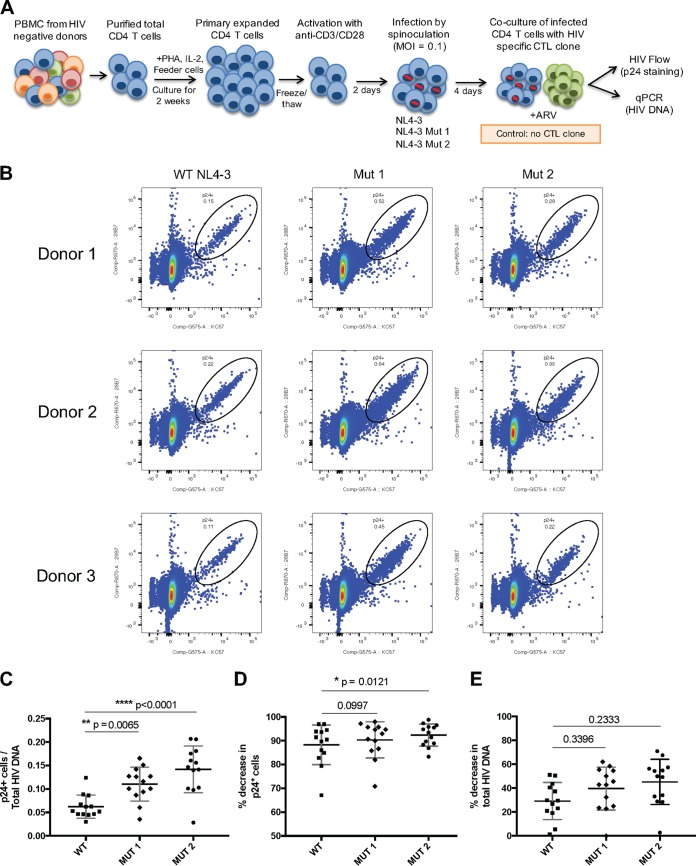
Primary CD4^+^ T cells infected with dCA-resistant viruses express more HIV antigens and are more susceptible to CTL-mediated killing. (A) Experimental design of the CTL killing assay in which target cells are isolated from CD4^+^ T cells purified out of PBMCs from HIV-negative donors and expanded in culture for 2 weeks, followed by activation with anti-CD3 and anti-CD28 antibodies and *in vitro* infection by spinoculation with wild type NL4-3 virus or dCA-resistant viruses. The viruses are allowed to grow in the CD4^+^ T cell culture for 4 days, and the killing assay is performed by coculturing these cells with HIV-specific CD8^+^ T cell clone in the presence of ARVs. The killing efficiency is calculated by the percent decrease in p24-expressing cells (assessed by flow cytometry) or HIV DNA (assessed by RT-qPCR) after the coculture compared to a control incubated without CTL clone. PHA, phytohemagglutinin; MOI, multiplicity of infection. (B) Representative plots of p24-expressing cells after 3 days of *in vitro* infection with wild-type NL4-3 virus and dCA-resistant viruses in primary human CD4^+^ T cells from three different donors. (C) Ratio between frequency of p24-expressing cells and total HIV DNA is significantly higher in cells infected with dCA-resistant viruses than in cells infected with wild-type NL4-3 virus. (D) CTL killing results showing percent decrease in frequency of CD4^+^ T cells expressing p24 after coculture with HIV-specific CTL clone. (E) CTL killing results showing percent decrease in total HIV DNA from CD4^+^ T cells after coculture with HIV-specific CTL clone. Data in panels C to E are presented as means ± SEM (*n* = 13). Paired ANOVA (Friedman’s test) was used for statistical comparison.

Given the higher transcriptional potential of the dCA-resistant viruses, we investigated whether it would result in higher CTL-mediated killing of infected CD4^+^ T cells. For that, we infected primary CD4^+^ T cells isolated from three different individuals with WT, MUT1, or MUT2 virus and cocultured these cells with HIV-specific CTL clones (Fig. 4A). The virus was allowed to replicate in the culture for 4 days after infection prior to the coculture and showed increased frequency of p24 expression in the cells infected with MUT1 and MUT2 viruses compared to cells infected with WT virus (Fig. 4B). The increased frequency in cells expressing virus antigens was not accompanied by increased levels of HIV DNA, as the ratio between the frequency of cells expressing p24 and cells harboring HIV DNA was significantly higher for MUT1 and MUT2 viruses than for the WT virus (Fig. 4C), confirming that higher fitness of the mutant viruses was due to heightened transcriptional rates. MUT2 seems to produce more viral capsid per cell, possibly explaining why it leads to higher cytotoxicity than MUT1 as observed in Fig. 3F to H. After coculturing the infected CD4^+^ T cells with an HIV-specific CTL clone, we assessed the level of CTL-mediated killing by the decrease in frequency of p24-expressing cells and total HIV DNA calculated based on infected cells cultured without the CTL clone. CTL-mediated killing resulted in a higher decrease of p24-expressing cells when infected with the mutant viruses than cells infected with the WT virus, with a significant difference for the MUT2 virus (Fig. 4D). The difference in HIV DNA levels was less prominent but also showed a trend toward higher decrease in cells harboring HIV DNA when infected with the mutant viruses than the cells infected with WT virus. These results confirmed that the mutant viruses express more antigens due to their elevated transcriptional levels that render the infected cells more susceptible to CTL killing.

### Generation of molecular clones of resistant viral isolates and chimeras to characterize dCA-resistant mutations.

**FIG 5:**
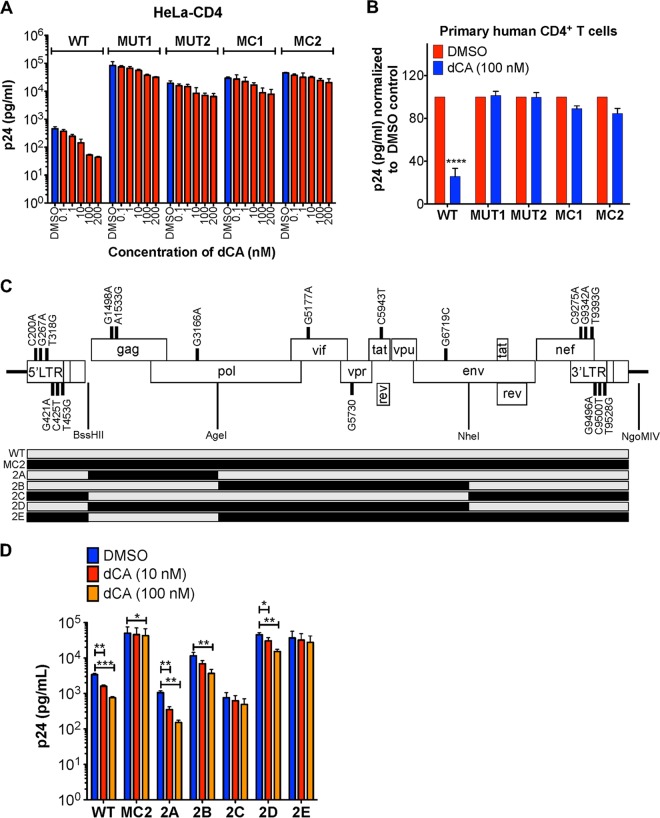
Molecular clones mimic the dCA resistance of natural isolates. (A) HeLa-CD4 were infected with WT, MUT1, or MUT2 virus or with the molecular clones (MCs) of dCA-resistant viruses (MC1 or MC2). Cells were than washed and treated with the indicated concentrations of dCA or DMSO. Capsid p24 was measured 48 h posttreatment using p24 ELISA (*n* = 2). (B) MCs and viral isolates are resistant to dCA in primary human CD4^+^ T cells isolated from three donors. CD4^+^ T cells were infected and treated with DMSO or 100 nM dCA. Capsid p24 was measured by ELISA 6 days after treatment. Data are normalized to the values for 100% DMSO control for each virus. See Fig. S4 for raw data (*n* = 3). (C) MC2 chimeras and resistance to dCA. Schematics of recombinant MC2 chimeras. (Top) Molecular chimeras were made based on MC2, and mutations and restriction enzyme sites are indicated. (Bottom) Schematic of the recombinant chimeras 2A to 2E made from MC2. The gray and black segments indicate the sequences from WT and MC2, respectively. (D) Susceptibility to dCA of the constructed chimeras. Chimeras 2C and 2E with mutations in Nef/LTR (as well as Vif, Vpr, Tat, and Env for 2E) are highly resistant to dCA. HeLa-CD4 cells were infected with the indicated viruses before adding dCA. Viral capsid production in the supernatant was measured 48 h later by p24 ELISA. All data are presented as means plus SEM. One-way ANOVA followed by a Bonferroni post-test were used for statistical comparisons in panel D. Statistical significance: *, *P* < 0.05; **, *P* < 0.01; ***, *P* < 0.001.

To study the roles of the mutations in MUT1 and MUT2 viruses, molecular clones 1 (MC1) and 2 (MC2) were synthesized using >50% consensus sequences. The complete sequences of MC1 and MC2 are available in Data Set S1 in the supplemental material. Viruses produced from MCs recapitulated the natural isolate’s resistance to dCA (Fig. 5A). Notably, all resistant viruses produced around 40- to 150-fold more viral particles than an equivalent inoculum of WT virus, again suggesting increased fitness. We also confirmed that both MUT viruses (MUT1 and MUT2) and MCs were resistant to 100 nM dCA in primary human CD4^+^ T cells (Fig. 5B and Fig. S4).

10.1128/mBio.01750-18.4 FIG S4 MCs and viral isolates are resistant to dCA in primary human CD4^+^ T cells. Primary human CD4^+^ T cells isolated from blood samples from three donors were infected overnight. Cells were then washed and treated with DMSO or 100 nM dCA. Capsid p24 was measured by ELISA 6 days after treatment. This figure shows the raw data of Fig. 5B. Download FIG S4, PDF file, 0.1 MB. Copyright © 2019 Mousseau et al. 2019 Mousseau et al. This content is distributed under the terms of the Creative Commons Attribution 4.0 International license.

10.1128/mBio.01750-18.6 DATA SET S1 MC1 and MC2 nucleotide sequences. The nucleotide sequences of the dCA-resistant molecular clones, MC1 and MC2, represent higher than 50% consensus sequences of nucleotide changes in MUT1 and MUT2, respectively. Download Data Set S1, PDF file, 0.03 MB. Copyright © 2019 Mousseau et al. 2019 Mousseau et al. This content is distributed under the terms of the Creative Commons Attribution 4.0 International license.

**FIG 6:**
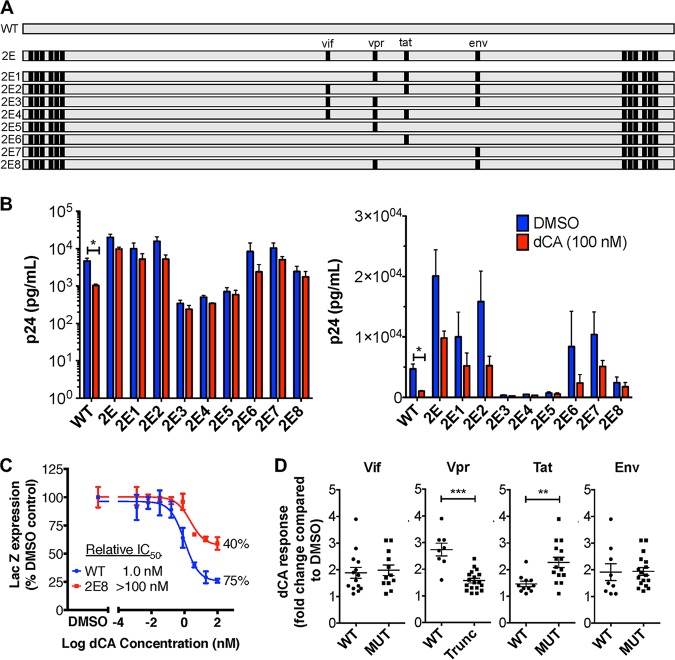
Characterization of the roles of Vif, Vpr, Tat and Env mutations in dCA resistance. (A) Schematics of site-specific sequence reversions from the dCA-resistant chimera 2E to that of the WT NL4-3 sequence. Nucleotide mutations in Vif, Vpr, Tat and Env were reverted individually to the WT sequence (2E1 to 2E4) or in combination (2E5 to 2E8). Gray squares show WT sequence; black squares show 2E mutations. (B) The molecular chimeras 2E5 and 2E8 with the Vpr truncation and six mutations in the Nef/LTR region confer the most resistance to dCA (*n* = 3, except for 2E7 [*n* = 2]). The experiment was performed as described in the legend to Fig. 5A. Logarithmic (left panel) and linear (right panel) representations of the data are shown. (C) CPRG assay of WT and 2E8 viruses with increasing concentrations of dCA. Relative IC_50_ is represented as well as the maximum inhibition upon dCA treatment. (D) Chimeras expressing a truncated Vpr are more resistant to dCA. Chimeras (2E to 2E8) were separated into groups containing either the WT or MUT protein for each viral protein. Each data point corresponds to the independent repeats from panel B. All data are presented as means ± SEM. The two-tailed paired *t* test and two-tailed Mann Whitney were used for statistical comparisons for panels B and D, respectively. Statistical significance: *, *P* < 0.05; **, *P* < 0.01; ***, *P* < 0.001. Trunc, truncated.

To define the roles of the mutations in the resistance to dCA, five chimeras were generated by combining different segments of WT and MUT2 genomes (Fig. 5C). We focused on mutations present in MUT2, since it contained all MUT1 changes (except for the NF-κB/Sp1 insertion in the LTR and an additional silent Tat mutation), plus five MUT2 unique mutations. The five constructed chimeras were named 2A (mutations in Gag and Pol), 2B (mutations in Vif, Vpr, Tat, and Env), 2C (mutations in Nef and LTRs), 2D (mutations present in 2A and 2B), and 2E (mutations present in 2B and 2C) (Fig. 5C). Viruses produced from these molecular chimeras were tested in parallel with the WT and MC2 viruses to assess their susceptibility to dCA (Fig. 5D). Virus 2A was shown to be as sensitive to dCA as the WT virus, indicating that mutations in Gag and Pol genes are not required for resistance to dCA. Resistance to dCA is comparable between MC2, 2C, and 2E viruses, further dismissing a possible role of the Gag and Pol mutations. Viruses 2B and 2D are partially resistant to dCA at 10 nM but inhibited at 100 nM dCA, suggesting that mutations in the Vif/Vpr/Tat/Env region may be important but need to be combined with mutations in the Nef/LTR region for complete resistance. Mutations contained in virus 2E and more specifically 2C seemed sufficient for resistance to dCA; however, the fitness of virus 2C is poor, not reflecting the high replicating capacity of MUT2. As such, we focused on chimera 2E to continue our studies and reversed mutations back to WT either individually or in combination (Fig. 6A). As expected, viruses with Vif, Vpr, Tat and Env mutations that were reverted individually back to WT (2E1 to 2E4) did not become totally sensitive to dCA (Fig. 6B). Nonetheless, 2E2 and 2E6, which express the full-length Vpr protein seemed the most affected by dCA (∼3-fold decrease between DMSO and dCA compared to 1.5- to 2-fold for 2E1, 2E3, 2E4, and 2E7) (Fig. 6B). Moreover, when Vif, Tat and Env mutations were reverted to WT and the Vpr mutation was still present (2E5), the virus appeared more resistant to dCA (1.2-fold difference between DMSO and dCA), suggesting a possible role of the truncated Vpr in dCA resistance (Fig. 6B). 2E8, which contains the Vpr truncation, alongside the Env mutation, has better fitness than 2E5 and shows 1.4-fold difference between DMSO and dCA (Fig. 6B). A LTR β-galactosidase reporter assay (CPRG) was used to measure the inhibitory dose-response curve of 2E8 upon dCA treatment (Fig. 6C). While still slightly affected by dCA, the observed inhibition of 2E8 plateaued at 40% versus 75% for the WT virus, as commonly observed (the 20 to 25% of inactivity corresponding to HIV-1 Tat-independent basal transcription) (18). To better define the impact of each viral protein in dCA resistance, viruses expressing either the WT or mutated form of each viral protein were compared (Fig. 6D). Vif and Env mutations do not seem to confer resistance to dCA. As suspected, the group of viruses containing the Vpr truncation (2E, 2E1, 2E3, 2E4, 2E5, and 2E8) are statistically more resistant to dCA. Interestingly, the mutation in the Tat gene, while not affecting the Tat protein sequence, seems to further sensitize viruses to dCA (1.5- versus 2.3-fold on average for the group of Tat WT versus MUT chimeras) (Fig. 6D). In sum, the Vpr truncation along with the six mutations in the Nef/LTR region seem to be required for resistance to dCA.

### Mechanism of action of mutations in HIV-1 LTR, Nef and Vpr genes in dCA resistance.

**FIG 7:**
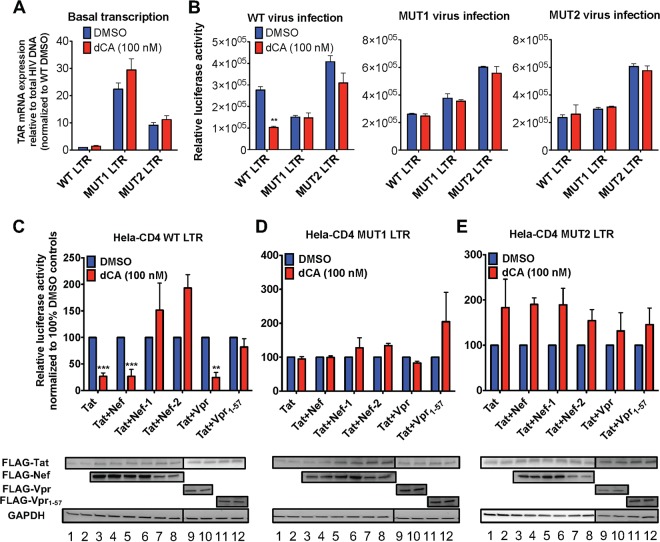
The LTRs of resistant viruses have enhanced transcriptional activity and largely contribute to dCA resistance. (A) Basal transcription of stable clones of HeLa-CD4 LTR promoters (WT, MUT1 and MUT2) driving luciferase. Twenty-four hours after plating, cells were treated with DMSO or 100 nM dCA for 48 h. TAR mRNA expression was measured and normalized to total proviral DNA. Data are normalized to 1 to the WT LTR-treated DMSO condition (*n* = 3). (B) Activation of WT or MUT LTRs by WT or dCA MUT viruses. HeLa-CD4 cells stably expressing WT or MUT LTR-Luc were infected for 16 h with WT or MUT viruses in the presence of DMSO or dCA (100 nM). Cells were washed and treated with dCA or DMSO, and luciferase activity was measured 48 h later (*n* = 3). (C to E) Effects of Tat, Nef and Vpr transactivation of WT and MUT LTRs in the presence or absence of dCA. (Top) Activity of the WT and MUT LTRs in the presence or absence of dCA after Tat transfection alone or in combination with Nef, Vpr or their mutated/truncated forms. At 24 h posttransfection, the cells were treated with either DMSO or 100 nM dCA for 48 h. Cells were then lysed and luciferase activity was measured. Data are normalized to 100% of the values for the DMSO controls (*n* = 3). (Bottom) Expression of FLAG-tagged Tat (FLAG-Tat), FLAG-Nef, FLAG-Nef-1, FLAG-Nef-2, FLAG-Vpr and FLAG-Vpr_1-57_ in the presence of DMSO or dCA from cells presented in the top panels, quantified by Western blotting using an anti-FLAG antibody and anti-GAPDH antibody as a control. All data are presented as means plus SEM. One-way ANOVA followed by a Bonferroni posttest were used for statistical comparisons. *, *P* < 0.05; **, *P* < 0.01; ***, *P* < 0.001; ****, *P* < 0.0001.

In an effort to understand the roles of mutations in the LTRs of the dCA-resistant viruses, we built vectors expressing firefly luciferase under the control of either the WT, MUT1, or MUT2 LTR and stably expressed them in HeLa-CD4 cells (HeLa-CD4 LTR-Luc cell) (Fig. 7A). Interestingly, the TAR mRNA expression from the basal activity of the MUT1 and MUT2 promoters, relative to total HIV DNA' was 10- to 30-fold higher than the WT (Fig. 7A). We then measured the reporter activity upon infection of the HeLa-CD4 LTR-Luc cells with the three viruses, with or without 100 nM dCA (Fig. 7B). In the cells containing the WT LTR, infection with WT virus resulted in 63% inhibition of the luciferase activity with dCA compared to the DMSO control (Fig. 7B, left panel). As suspected, the reporter activity upon infection with MUT1 and MUT2 viruses of HeLa-CD4 cells carrying either WT or MUT LTRs was indifferent to the presence of 100 nM dCA (Fig. 7B, middle and right panels). Strikingly though, luciferase expression after WT virus infection of MUT1 or MUT2 LTR-Luc reporter cells was not inhibited by dCA (Fig. 7B, left panel), suggesting that the mutations in the LTR are determining the resistance to dCA, even if, as shown above, other proteins, such as truncated Vpr, may also contribute to dCA resistance.

Since the mutations in the LTR encompass the Nef coding sequence, it was important to evaluate whether resistance to dCA was associated with a combination of activities, not only from the higher promoter transcriptional activity mediated by the mutations in the LTR but also from the mutations in the Nef protein itself (Table 1). As such, we measured WT or MUT promoter activity upon cotransfections of the WT Tat with WT Nef or mutated Nef-1 or Nef-2 plasmids containing the mutations present in MUT1 and MUT2 viruses, respectively (Fig. 7C to E). In parallel, we also investigated the ability of WT Vpr or truncated Vpr (Vpr_1-57_) to enhance promoter activity in combination with Tat. In the WT HeLa-CD4 LTR-Luc cells, overexpression of Tat alone or in combination with either WT Nef or Vpr transactivated the WT promoter and remained sensitive to dCA (Fig. 7C, top panel; see Fig. S5A for raw data). However, when Nef-1, Nef-2, or Vpr_1-57_ was coexpressed with Tat, transcription from the viral WT promoter was not inhibited by dCA. For Tat and Nef-1/Nef-2 cotransfections, transcription in the presence of dCA was even higher than the DMSO control. In MUT1 and MUT2 LTR-Luc cells, the Tat insensitivity to dCA was evident under all conditions (Fig. 7D and E, top panels; see Fig. S5B and C for raw data). In fact, results showed either no inhibition or a slight activation in the presence of dCA. By Western blot analysis, we verified similar expression of Tat, Nef, and Vpr proteins or their mutants in the presence of DMSO and dCA, ensuring that the differences in the luciferase activity were not due to changes in protein expression (Fig. 7C to E, bottom blots). Altogether, these results suggest that viral evasion to dCA results from a combination of mutations in the HIV-1 LTR, Nef, and Vpr proteins.

10.1128/mBio.01750-18.5 FIG S5 Effects of Tat, Nef and Vpr transactivation of WT and MUT LTRs in the presence or absence of dCA. Activity of the WT and MUT LTRs in the presence or absence of dCA after Tat transfection alone or in combination with Nef or Vpr or their respective mutants. This figure shows the raw data of Fig. 7C to E (*n* =3). All data are presented as mean ± SEM. Download FIG S5, PDF file, 0.1 MB. Copyright © 2019 Mousseau et al. 2019 Mousseau et al. This content is distributed under the terms of the Creative Commons Attribution 4.0 International license.

### HIV-1 Nef and Vpr boost NF-κB activation.

**FIG 8:**
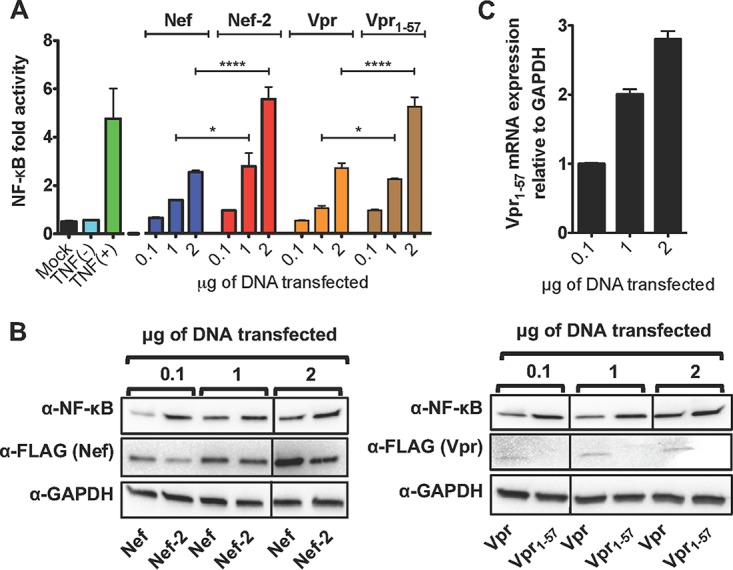
Impact of Nef mutation and Vpr truncation on NF-κB activation. (A) HeLa-CD4 cells were cotransfected with a luciferase reporter vector under the control of a NF-κB promoter, with increasing amounts of Nef, Nef-2, Vpr or Vpr_1-57_ expression vectors. Luciferase activity was measured 48 h posttransfection. Renilla luciferase construct was cotransfected for normalization. For a control for NF-κB activation, the cells were treated or not treated with TNF-α for 30 min prior to lysis (*n* = 3). (B) Representative Western blot of proteins transfected in panel A revealed with anti-FLAG (α-FLAG), anti-NF-κB or anti-GAPDH antibodies. Expression levels of Vpr_1-57_ were too low to be detected by Western blot (*n* = 3). (C) Detection by RT-qPCR of mRNA levels of Vpr_1-57_ from samples indicated in panel A (*n* = 2). All data are presented as means plus SEM. The two-way ANOVA followed by a Bonferroni post-test were used for statistical comparisons. *, *P* < 0.05; ****. *P* < 0.0001.

Nef has been known to activate the NF-κB pathway and Vpr to activate LTR expression (for a review, see reference 25). As such, we investigated whether the mutant Nef and truncated Vpr might differentially activate the NF-κB pathway by expressing Nef and Vpr variants in HeLa-CD4 cells carrying an NF-κB promoter driving luciferase expression (Fig. 8). A transfection of 0.1 to 2 μg DNA of WT Nef and Vpr proteins increased NF-κB-driven luciferase expression in a dose-dependent manner up to 2.5-fold, while Nef-2 and Vpr_1-57_ proteins resulted in 2- to 5-fold increases (Fig. 8A). We confirmed by Western blotting and real-time quantitative PCR (RT-qPCR) that the differences in transcriptional activation were not due to differential protein/RNA expression (Fig. 8B and C). Together, these results suggest that mutated Nef and Vpr_1-57_ have the ability to potentiate NF-κB-driven transcription.

## DISCUSSION

In this study, we characterized the determinants of HIV-1 resistance to the Tat inhibitor dCA. Escape variants were identified after a 12-month period of passaging HIV-1 NL4-3 in HeLa-CD4 cells. Evolution of resistance to dCA was also attempted using T-lymphocytic cell lines such as CEM-SS and Jurkat; unfortunately, upon dCA treatment, the virus was consistently lost, not allowing reinfection of naive cells. Viruses usually develop resistance to ARVs within 6 to 30 weeks, highlighting the higher genetic barrier to escape a Tat inhibitor (26–29). The nucleotide sequence landscape of the two identified dCA-resistant HIV-1 isolates includes changes in the LTR, Vpr (truncation), and Nef required for total evasion to dCA, again reinforcing the high genetic barrier to dCA resistance.

Interestingly, mutations in Tat or TAR were not detected. dCA binds to the basic domain of Tat (18), which is extremely conserved across viral clades. Mutations in Tat and TAR have been shown to dramatically affect viral replication (30–32). To escape pressure from a Tat inhibitor, it was somewhat expected that on one hand we observe changes that would promote Tat-independent transcription, and on the other hand, increased viral transcription with augmented Tat production to quench dCA. As such, six mutations were found in the U3 region of the LTR, three in the modulatory region of the promoter and three in the core region upstream of the transcription start site, with one major insertion of two NF-κB/one Sp1 binding site between the Sp1 sites II and III in MUT1 (Fig. 2B). The correlation between LTR variations and differential promoter activity has been well documented (33–38).

The C425T mutation, identified in both resistant viruses, is position 1 of the HIV-1 TATA box and changes the motif CATATAA to TATATAA, previously shown to slightly increase viral fitness (Table 1 and Fig. 2B) (39). This mutation was reported in a study of LTR-defective viruses carrying one or two NF-κB binding sites but lacking the three Sp1 sites that were passaged for more than a month in CEM cells or PBMCs (40). An additional T423A mutation appeared in these PBMC-grown viruses, which is 2 nucleotides upstream of the key MUT2 G421A, possibly reflecting a role for this error-prone region upstream of the TATA box in counteracting transcriptional repression mechanisms. Of note, we individually mutated each of the six mutations in the LTR of chimera 2E and assessed their susceptibility to dCA. Unfortunately, we could not confidently pinpoint a particular nucleotide, suggesting that the combination of the six mutations might be required. Also, we cannot exclude the possibility that these mutations, which are also present in the 3′ LTR, impact the mRNA splicing and polyadenylation machinery, giving a positive replication advantage against dCA.

Another important contribution to the viral evasion to dCA is the C-terminal region truncation of the viral protein Vpr (Vpr_1-57_), demonstrated using virions expressing truncated Vpr (Fig. 6) and Tat-dependent transactivation upon cotransfections (Fig. 7). Our results suggest a mechanism of resistance based on potentiation of basal transcription following an increase in NF-κB activation by Vpr_1-57_ (Fig. 8). The activity of Vpr in modulating NF-κB transduction pathway has been debated (41–44). Vpr may stimulate the HIV-1 LTR promoter via NF-κB and AP-1 signaling by activating TAK1 and the MKK pathways (43). A point mutation in Vpr (Ser79Ala) results in loss of TAK1 binding and promoter activation, likely ruling out the use of this pathway by Vpr_1-57_ (43). Vpr was also shown to upregulate HIV-1 transcription by binding Sp1 on the 5′ LTR (45) and to cooperate with Tat and cyclin T1 to activate transcription (46). Other studies backed a mechanism where Vpr would play an indirect role in HIV-1 transcription (25, 47, 48) by inducing G_2_ arrest, a cell cycle phase where HIV transcription is the most active (25, 47, 49). Both the third helix and C-terminal residues of Vpr promote G_2_ arrest indirectly increasing HIV transactivation (49), and Vpr Arg80Ala impairs G_2_ arrest function and reduced HIV-1 transcription (50–52). It is thus unlikely that Vpr_1-57_ mediates G_2_ arrest, promoting Tat-independent activity. Interestingly, truncated Vpr proteins are often observed in cultured viruses *in vitro*, as it facilitates stable persistence of viral expression in chronically infected cells (53, 54). *In vivo* though, Vpr is well conserved in both HIV-1-infected humans and simian immunodeficiency virus (SIV)-infected monkeys (reviewed in reference 49); in fact, truncated Vpr viruses reverted back to wild type after 2 to 3 years of infection in both chimpanzees and infected laboratory workers (47), emphasizing its role in virus replication and pathogenesis. Thus, we cannot rule out the possibility that Vpr_1-57_ is an *in vitro* phenomenon only that would unlikely occur *in vivo*, favoring an alternate escape mechanism. Future studies will explore whether Vpr_1-57_ activates NF-κB by interfering with NF-κB and IκB interaction or whether it results from indirect activity on a cellular gene.

Our results demonstrate that mutations in the Nef protein also contribute to resistance to dCA, namely, Ser163Arg (C9275A), Asp186Asn (G9342A), and Phe203Val (T9393G) (Table 1). All three amino acids/nucleotides are well conserved among the 4,146 sequences of the major subtypes (https://www.hiv.lanl.gov/content/sequence/ANALYZEALIGN/analyze_align.html). Either a Cys (62%) or Ser (30.2%) can be found in Nef at position 163, but rarely an Arg (1.9%). The amino acid at position 186 is almost always an Asp (95.7%), followed by an Asn mainly present in clade D, as found in MUT2. Finally, the amino acid at position 203 is 99.5% of the time either a Tyr (90.1%) or a Phe (9.4%). To our knowledge, none of these amino acids have been found necessary for Nef activity. Nef Ser163Arg, common to both Nef MUT1 and MUT2, is in the dileucine motif ENTSLL found in NL4-3 within the C-terminal flexible loop (positions 149 to 179) needed for AP-2 binding and CD4 downregulation (55). However, mutation of this residue did not impair Nef/AP-2 binding or CD4 downregulation (56, 57). Ser163 is important to bind and stabilize AP-3 (and to a lesser extent AP-1) (58). The second Nef mutation Asp186Asn is part of the D186-x-x-x-x-(F/Y)191 β-catenin binding motif (59), which is especially conserved in clade B isolates (92.7%) such as NL4-3 (59). Nef has been reported to inhibit the Wnt/β-catenin/TCF-4 signaling pathway, possibly by competing with TCF-4/LEF1 binding sites (59). In this study, Nef Asp186Ala abrogated binding to wild-type β-catenin (59). A recent study showed that Nef Asp186Ala impaired CD4 downregulation, actin dynamics, and cell motility (60). It is thus possible that Nef Asp186Ala competes with Wnt/β-catenin/TCF-4 signaling, helping recruitment of C/EBP and NF-κB to promote HIV-1 transcription. Finally, the third mutation in Nef, Phe203Val, located in the highly conserved C-terminal tyrosine motif Tyr202-(Tyr/Phe)203 likely impairs CD4 downregulation (61). Like Vpr protein, Nef is also a multitasker protein involved in various functions such as activation of NF-κB, AP-1, and JNK and thus transactivation of the HIV-1 LTR. A recent report shows that HIV-1 Nef from most HIV strains boosts IKK-β-mediated NF-κB activation (62). Our results suggest that mutations in Nef proteins of dCA-resistant viruses trigger higher NF-κB nuclear translocation enhancing Tat-independent HIV transcription. The direct relationship between NF-κB recruitment and RNAPII elongation via P-TEFb recruitment has been previously established (63). Future studies will investigate the relationship between the identified Nef variants with IKK-β degradation.

Collectively, our study suggests that the combined effects of mutations in the LTR, Nef and Vpr result in superior viral fitness and evasion to dCA, emphasizing the high genetic barrier to the development of resistance to dCA. However, it is uncertain whether these mutant viruses would have been identified *in vivo*. Their increased transcription fitness, confirmed by their augmented recruitment of RNAPII to the promoter (Fig. 3I), may ultimately be detrimental. The inability of these variants to control their entry into latency may lead to high cytopathic effects (Fig. 3F to H), and possibly to clearance by the immune system. We have shown that *in vitro*, the resistant viruses express higher levels of viral proteins and are more susceptible to CTL-mediated killing (Fig. 4). Future work will detail the molecular mechanisms by which MUTs (MUT1 or MUT2) Nef and Vpr regulate NF-κB, as well as the landscape of transcription factors associating with mutant LTRs, which may reveal novel aspects of HIV-1 transcriptional regulation.

## MATERIALS AND METHODS

### Plasmids.

pNL4-3 was obtained through the NIH AIDS Reagent Program. ACGT Inc. synthesized the pET28b-NL4-3-MUT1 and pET28b-NL4-3-MUT2 vectors. The LTRs were cloned using XhoI and HindIII restriction sites into pGL4.22[*luc2CP*/Puro] (Promega) to obtain, respectively, pGL4.22_WT LTR-Luc, pGL4.22_MUT1 LTR-Luc and pGL4.22_MUT2 LTR-Luc. pUHD10-3 CDK11p110(c-FLAG) (a gift from Jill Lahti, St. Jude Children Hospital) and pCDNA4/TO/myc-His B (Thermo Fisher Scientific) were used to subclone parts of the WT and MUT viruses (MUT1 and MUT2) to facilitate mutagenesis (see Tables S1 and S2 in the supplemental material).

10.1128/mBio.01750-18.7 TABLE S1 Cloning strategy. Each donor vector was digested with the indicated restriction enzymes, and the released fragment containing the insert was cloned into the acceptor vector. The nomenclature is based on pNL4-3 numbering. Download Table S1, PDF file, 0.05 MB. Copyright © 2019 Mousseau et al. 2019 Mousseau et al. This content is distributed under the terms of the Creative Commons Attribution 4.0 International license.

10.1128/mBio.01750-18.8 TABLE S2 Two-step mutagenesis and cloning strategy. Mutagenesis was performed on the parent plasmid with the indicated primers. The newly mutated plasmid was digested with the indicated restriction enzymes and cloned into the acceptor vector. Download Table S2, PDF file, 0.04 MB. Copyright © 2019 Mousseau et al. 2019 Mousseau et al. This content is distributed under the terms of the Creative Commons Attribution 4.0 International license.

MDH1-PGK-FLAG-Tat86 was obtained by PCR amplification of the 3X-FLAG-Tat86 from pFI-FLAG-Tat(86) WT using primers ^5′^CCGCCACCAT GGACTACAAA^3′^ and ^5′^AATGTCGACC TACTGTTCCT TCGGGCCTGT CG^3′^, and cloned into MDH1-PGK-GFP_2.0 with NcoI/SalI enzymes. MDH1-PGK-FLAG-Nef/-1/-2 were obtained by PCR amplification from pNL4-3, pET28b-NL4-3-MUT1, and pET28b-NL4-3-MUT2, respectively, using primers ^5′^GACAAGCTTG CGGCCGCATG GGTGGCAAGT GGTCAAAAAG^3′^ and ^5′^CTTGGCTGCA GGTCGACCTA TATCAGCAGT TCTTGAAGTA CTCC^3′^ and cloned into the MDH1-PGK-FLAG-Tat with NotI/SalI enzymes. Similarly, MDH1-PGK-FLAG-Vpr/Vpr_1-57_ were generated by PCR amplification using primers ^5′^GACAAGCTTG CGGCCGCAAT GGAACAAGCC CCAGAAG^3′^ and ^5′^CTTGGCTGCA GGTCGACCTA TACTAGGATC TACTGGCTCC^3’^. We inserted a stop codon for the construct Vpr_1-57_ using primers ^5′^CTTGGGCAGG AGTGAAGCCA TAATAAG^3’^ and ^5′^CTTATTATGG CTTCACTCCT GCCCAAG^3’^.

### Mutagenesis.

Mutagenesis was performed with the KOD Hot Start DNA polymerase kit (Toyobo Co., Ltd.). The following cycling conditions were used: an initial denaturation (95°C for 2 min), followed by 18 cycles of amplification (one cycle consisting 95°C for 50 s, 60°C for 50 s, and 68°C at 1 min/kb), a final elongation step (68°C at 2 min/kb) and hold at 4°C. The DNA was then treated with DpnI (NEB) for 1 h at 37°C, and the DNA was then cleaned by PCR and transformed in Escherichia coli Stellar competent cells or XL-10 Gold cells. Colonies were then grown, purified, and sent for sequencing (Eurofins Genomics). Primers are summarized in Table S2.

### Cells.

CEM-SS and Jurkat cells were obtained from the American Type Culture Collection (ATCC). Primary CD4^+^ T cells were isolated as previously described (21). Primary CD4^+^ T cells were infected with 100 ng of WT NL4-3 or mutant viruses. Approximately 16 h later, the cells were washed and treated with dimethyl sulfoxide (DMSO) or 100 nM didehydro-cortistatin A (dCA) for 6 days. Supernatants were collected, and p24 was measured by ELISA (catalog no. 5447; Advanced BioScience Laboratories, Inc.). To obtain chronically infected Jurkat populations, cells were infected at a multiplicity of infection of 0.5 for 12 h. Cells were washed three times. Fresh culture medium was then added, and the cells were passaged twice a week for 3 months. CEM-SS and Jurkat cells cultured in Roswell Park Memorial Institute 1640 (RPMI 1640) medium with 10% fetal bovine serum (FBS) and PSG (penicillin [100 U/ml], streptomycin [100 g/ml], and l-glutamine [2 mM]). HeLa-CD4 (HT4-6C) cells were obtained from the NIH AIDS Reagent Program. Stable HeLa-CD4 LTR-Luc cells were obtained by transfecting HeLa-CD4 cells with pGL4.22_WT LTR-Luc, pGL4.22_MUT1 LTR-Luc, and pGL4.22_MUT2 LTR-Luc and selected with puromycin (2 μg/ml). HeLa-CD4 cells were cultured in Dulbecco’s modified Eagle’s medium supplemented with 5% FBS and PSG. All cells were cultured at 37°C and 5% CO_2_.

### Generation of dCA-resistant mutants.

HeLa-CD4 cells (2 × 10^5^) were infected with 250 ng of p24 for 6 h. Cells were then washed, and DMSO or 0.1 nM dCA was added to the infected cells and incubated for 72 h. Thereafter, 1/10th of the supernatant harvested from the cells treated with DMSO and half of the supernatant from the cells treated with dCA were used to infect HeLa-CD4 cells for 24 h. The next day, the cells were washed, drugs were added, and the cells were incubated for 72 h. The levels of p24 from the collected viral supernatants were measured by ELISA to normalize the input upon infection and assess signs of resistance to dCA. Viruses were passaged by acute infection, each week for about 12 months, in increasing concentrations of dCA, until viruses could replicate in 1 μM dCA.

### Deep sequencing of the viral genome.

CEM-SS cells were used to amplify viruses. The viral genomic RNA of the three isolates were extracted from the viral particles using the QIAamp viral RNA minikit (Qiagen) and sent to ACGT, Inc., which performed the cDNA synthesis and whole-genome population sequencing, using Next Generation Sequencing Illumina technology. Molecular clones of the two dCA-resistant viral isolates were synthesized and cloned in the vector pET28b to obtain pET28b-NL4-3-MUT1 and pET28b-NL4-3-MUT2.

### Cloning of the chimeras.

Each vector was digested with the indicated restriction enzymes (NEB), and the released fragment containing the insert was cloned by ligation into the dephosphorylated vector (Table S1). Briefly, all chimeras were made by using fragments of pET28b-NL4-3-MUT2 cloned into the pNL4-3 vector, using combinations of unique restriction sites. Desalted ligated products were transformed into Escherichia coli Stellar or XL10-Gold cells (Agilent). Colonies containing the chimeras were grown, purified (Qiagen) and sequenced (Eurofins Genomics).

### Infection experiments.

HeLa-CD4 cells were infected with the indicated concentrations of viruses normalized with the p24 amount, for the indicated time. Fresh culture media containing DMSO or compounds were then added to the infected cells. At 48 to 96 h after infection, viral supernatants were collected and p24 was assessed by ELISA.

CEM-SS cells were infected, with 600 ng of p24, for 12 h. Cells were washed and treated with DMSO or 500 nM dCA. At 72 h postinfection, viral supernatants were collected, and p24 was assessed by ELISA.

Jurkat cells were infected, with 600 ng of p24, for 12 h. Cells were washed and split every 4 days until chronic infection was observed and monitored by p24 ELISA. Chronically infected Jurkat cells were treated with 100 nM dCA or raltegravir for 4 days, and p24 was assessed by ELISA.

Primary CD4^+^ T cells were infected at a multiplicity of infection of 0.1 by spinoculation at 2,000 rpm for 1 h at 37°C. Cells were maintained in RPMI 1640 medium containing 8% human serum, 10 ng/ml of recombinant human interleukin-2 (rhIL-2), 5 BRMP U/ml of natural human interleukin-2/TCGF, and 100 U/ml of penicillin-streptomycin. Every 3 or 4 days, cells were counted in an automated cell counter with trypan blue staining, and 500,000 cells were removed for flow cytometry analysis, followed by a half-medium change.

### Total DNA extraction and RT-qPCR analysis.

Total genomic DNA and integrated proviruses were quantified as previously described (21).

### mRNA extraction and RT-qPCR analysis.

Total mRNA was extracted and analyzed as previously described (21). Primer details are in supplemental information (Table S3) or in reference 19.

10.1128/mBio.01750-18.9 TABLE S3 Primers used to analyze the DNA immunoprecipitated by RNAPII ChIP. Download Table S3, PDF file, 0.04 MB. Copyright © 2019 Mousseau et al. 2019 Mousseau et al. This content is distributed under the terms of the Creative Commons Attribution 4.0 International license.

### Basal expression of HeLa-CD4 LTR-Luc cell lines.

Stable HeLa-CD4 WT, MUT1 or MUT2 LTR-Luc cells were treated with DMSO or 100 nM dCA. At 48 h posttreatment, TAR mRNA expression was measured and normalized to the number of integrated provirus.

### Infection of stable HeLa-CD4 LTR-Luc cells.

Stable HeLa-CD4 WT, MUT1, or MUT2 LTR-Luc cells were infected, with 150 ng of p24, for 16 h in the presence of the drug. Cells were washed and treated. At 48 h after treatment, the cells were lysed and luciferase activity was measured as previously shown (19, 64).

### Western blot analysis.

Protein extracts from the luciferase assay and NF-κB assay were normalized using Bradford assay and analyzed by SDS-PAGE followed by Western blotting. Anti-FLAG antibody (catalog no. F3165; Sigma) at 1:5,000 dilution, anti-NF-κB antibody (catalog no. 3033: CST) at 1:1,000 dilution, and anti-glyceraldehyde-3-phosphate dehydrogenase (anti-GAPDH) antibody (catalog no. SC-32233; Santa Cruz) at 1:10,000 dilution were used.

### Transfection of stable HeLa-CD4 LTR-Luc cells.

HeLa-CD4 cells stably expressing the WT, MUT1, or MUT2 LTR-Luc were transfected with 1.0 μg of MDH1-PGK-FLAG-Tat86 with either 1.0 μg of MDH1-PGK-GFP_2.0 or MDH1-PGK-FLAG-Nef/-1/-2, MDH1-PGK-FLAG-Vpr/Vpr_1-57_. At 24 h posttransfection, the cells were treated for 48 h, and luciferase activity was measured as described above.

### Luciferase expression driven by an NF-κB promoter.

HeLa-CD4 cells were cotransfected with 1.0 μg of NF-κB-Luc (catalog no. E8491; Promega) and 0.1 μg of *Renilla* luciferase with MDH1-PGK-FLAG-Nef/Nef-2, MDH1-PGK-FLAG-Vpr/Vpr_1-57,_ using *Trans*IT*-*293 transfection reagent (*Mirus*) per the manufacturer’s instruction. Cell were lysed 48 h later, with 1× Lysis Buffer and luciferase activity was measured with Promega Dual-Luciferase Reporter Assay per the manufacturer’s protocol. For a control, cells were treated with TNF-α (20 ng/ml) for 30 min before lysis.

### Chromatin immunoprecipitation (ChIP) assay.

The ChIP assay was performed, as previously described, with small changes, detailed below (21, 22). Briefly, HeLa-CD4 cells acutely infected with WT, MUT1 and MUT2 viruses and treated with DMSO or dCA (100 nM) were passaged every 3 days. At day 9, cells were cross-linked. ChIP samples (5 μl) or input 10% DNA was used in 20-μl RT-qPCR mixtures with primers listed in Table S3. Input (1%) was used to standardize the values obtained.

### β-Galactosidase (CPRG) assay.

The CPRG assay was performed as previously described (18, 19).

### Evaluation of cell viability by flow cytometry.

HeLa-CD4 cells acutely infected with the three viruses were stained with the APC-labeled antibody to annexin V (catalog no. 640919) and the Live/Dead staining Zombie Red (catalog no. 423109) for 15 min at 4°C, and then fixed with 2% formaldehyde at room temperature for 15 min. Samples were analyzed using a BD Accuri C6Plus flow cytometer, followed by FlowJo software. Live and dead cells were resolved in the FL2 (Zombie Red) and FL4 (annexin V) flow channels.

Primary CD4^+^ T cells infected with WT, MUT1 and MUT2 viruses were stained with Fixable Aqua Live/Dead (Invitrogen) for 10 min at room temperature and with anti-CD3 APC, anti-CD4 BV650, and anti-CD8 VB785 (Biolegend) for 20 min at 4°C and then fixed with 2% formaldehyde. Samples were analyzed in a LSRII (BD Biosciences), and data were analyzed with FlowJo v10 (FlowJo, LLC).

### CTL-mediated killing assay.

Primary CD4^+^ T cells were purified from PBMCs of HIV-negative donors and expanded in culture for 2 weeks, followed by activation with anti-CD3 and anti-CD28 antibodies and *in vitro* infection by spinoculation (centrifugation at 2,000 rpm for 1 h at 37°C) with wild-type NL4-3 virus or dCA-resistant viruses. The viruses were allowed to grow in culture for 4 days, and the killing assay was performed by coculturing these cells with HIV-specific CTL clone in the presence of ARVs. After the coculture, the killing efficiency was calculated compared to a control incubated without CTL clone by the percent decrease in p24-expressing cells assessed by flow cytometry as previously described (65) and by the percent decrease in HIV DNA assessed by RT-qPCR as previously described (66).

### Statistical analysis.

The *P* values were calculated using either the two-way analysis of variance (ANOVA) or one way followed by a Bonferroni *post hoc* test. The two-tailed paired *t* test and two-tailed Mann-Whitney test were used when required. *P* values of <0.05 were considered statistically significant. Statistical analysis and data representation were performed using the GraphPad Prism software (San Diego, CA, USA).

### Ethics statement.

Primary human CD4^+^ T cells were collected from blood samples from deidentified seronegative subjects and purchased from OneBlood–Florida.
