# Supplementary figures and images for: Resistance to the Tat Inhibitor Didehydro-Cortistatin A Is Mediated by Heightened Basal HIV-1 Transcription
Source: mBio. 2019 Jul 2;10(4):e01750-18. doi: 10.1128/mBio.01750-18 (PMC6606815; doi:10.1128/mBio.01750-18)

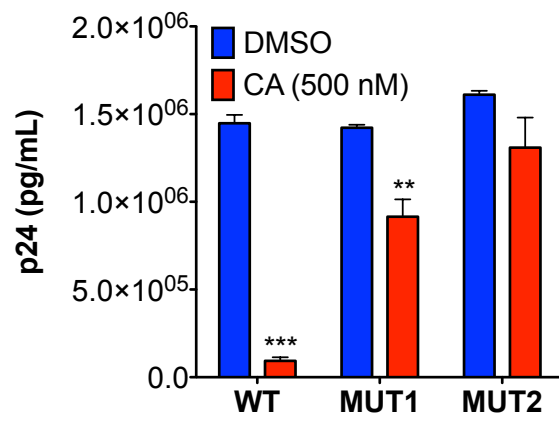

Supplement: FIG S1 [file mBio.01750-18-sf001.pdf]

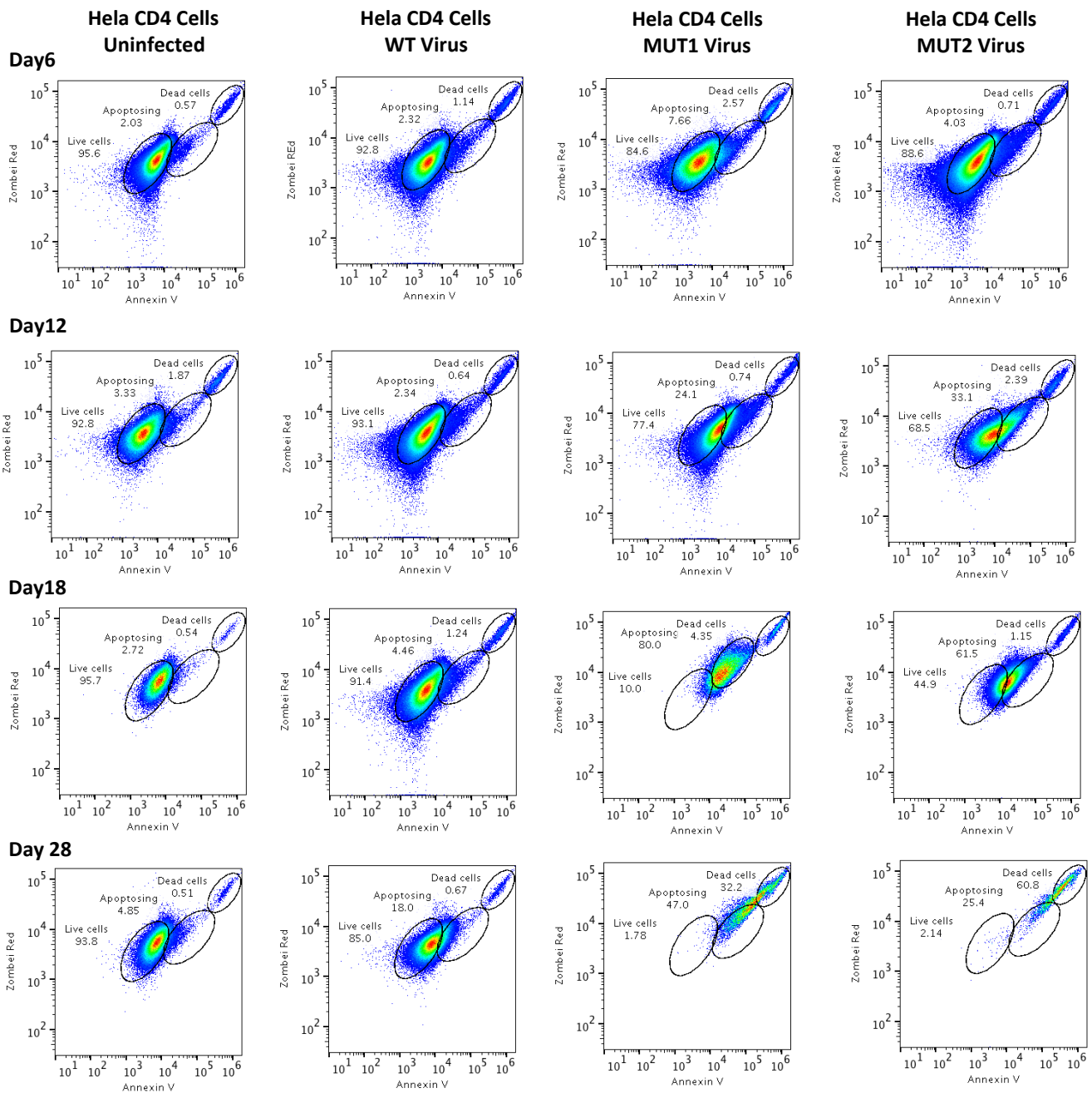

Supplement: FIG S2 [file mBio.01750-18-sf002.pdf]

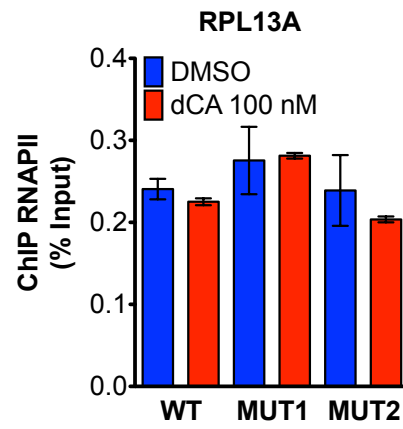

Supplement: FIG S3 [file mBio.01750-18-sf003.pdf]

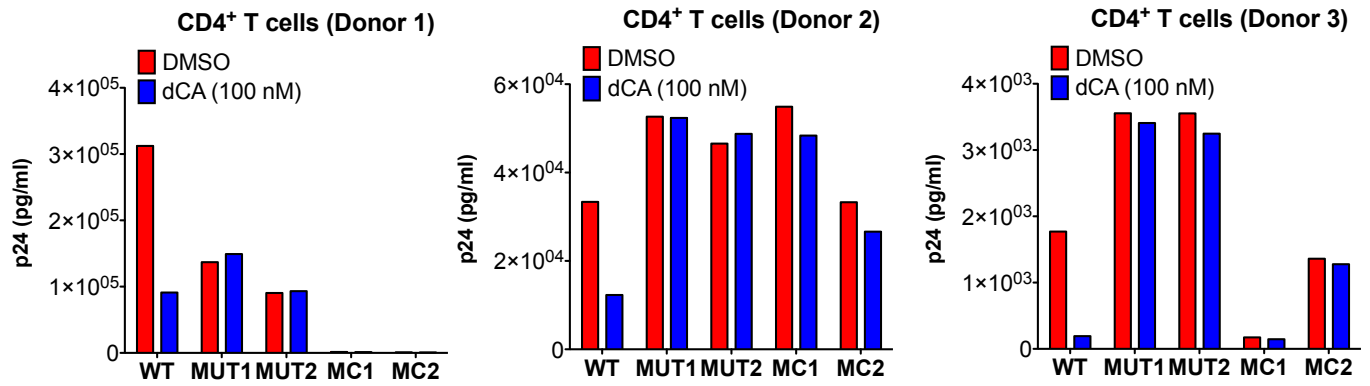

Supplement: FIG S4 [file mBio.01750-18-sf004.pdf]

**A**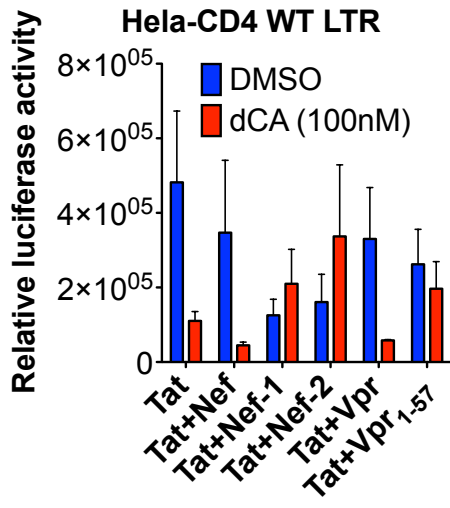**B**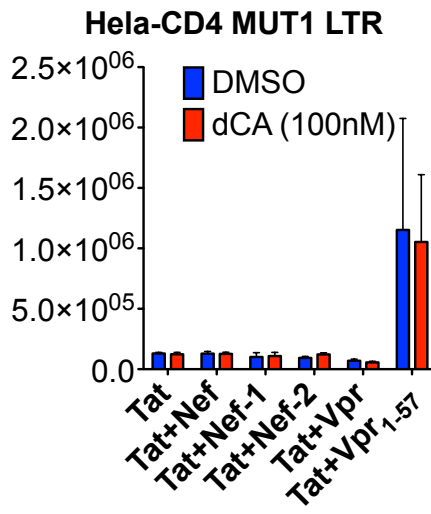**C**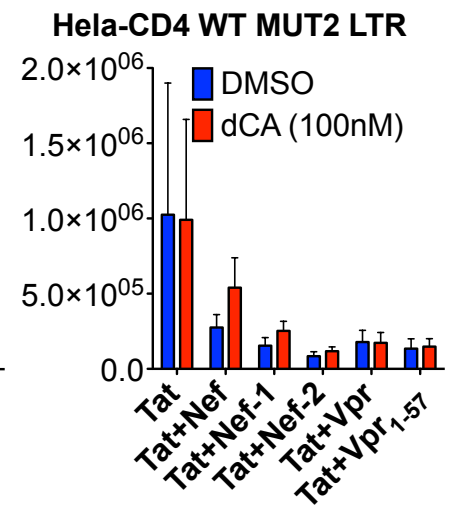

Supplement: FIG S5 [file mBio.01750-18-sf005.pdf]
