# Supplementary material for: Resistance to the Tat Inhibitor Didehydro-Cortistatin A Is Mediated by Heightened Basal HIV-1 Transcription
Source: mBio. 2019 Jul 2;10(4):e01750-18. doi: 10.1128/mBio.01750-18 (PMC6606815; doi:10.1128/mBio.01750-18)
Supplement: DATA SET S1 [file mBio.01750-18-sd001.pdf]

MC1

TGGAAGGGCTAATTTGGTCCCAAAAAAGACAAGAGATCCTTGATCTGTGGATCTACCACACACAAGGCT
ACTTCCCTGATTGGCAGAACTACACACCAGGGCCAGGGATCAGATATCCACTGACCTTTGGATGGTGCTT
CAAGTTAGTACCAGTTGAACCAGAGCAAGTAGAAGAGGCCAATGAAGGAGAGAACAACAGATTGTTAC
ACCCTATGAGCCAGCATGGGATGGAGGACCCGGAGGGAGAAGTATTAGTGTGGAAGTTTGACAGCCTC
CTAGCATTTCGTCACATGGCCCGAGAGCTGCATCCGGAGTACTACAAAGACTGCTGACATCGAGCTTTCT
ACAAGGGACTTTCCGCTGGGGACTTTCCAGGGAGGTGTGGCCTGGTGCTACAAGGAACTTTCCGCTGG
GGACTTTCCAGGGAGGCGTGGCCTGGGCGGGACTGGGGAGTGGCGAGCCCTCAGATGCTATATATAAG
CAGCTGCTTTTTGCCTGTACGGGGTCTCTCTGGTTAGACCAGATCTGAGCCTGGGAGCTCTCTGGCTAAC
TAGGGAACCCACTGCTTAAGCCTCAATAAAGCTTGCCTTGAGTGCTCAAAGTAGTGTGTGCCCGTCTGTT
GTGTGACTCTGGTAACTAGAGATCCCTCAGACCCTTTTAGTCAGTGTGGAAAATCTCTAGCAGTGGCGCC
CGAACAGGGACTTGAAAGCGAAAGTAAAGCCAGAGGAGATCTCTCGACGCAGGACTCGGCTTGCTGAA
GCGCGCACGGCAAGAGGCGAGGGGCGGCGACTGGTGAGTACGCCAAAAATTTTGACTAGCGGAGGCT
AGAAGGAGAGAGATGGGTGCGAGAGCGTCGGTATTAAGCGGGGGAGAATTAGATAAATGGGAAAAA
ATTCGGTTAAGGCCAGGGGGAAAGAAACAATATAAACTAAAACATATAGTATGGGCAAGCAGGGAGCT
AGAACGATTCGCAGTTAATCCTGGCCTTTTAGAGACATCAGAAGGCTGTAGACAAATACTGGGACAGCT
ACAACCATCCCTTCAGACAGGATCAGAAGAACTTAGATCATTATATAATACAATAGCAGTCCTCTATTGT
GTGCATCAAAGGATAGATGTAAAAGACACCAAGGAAGCCTTAGATAAGATAGAGGAAGAGCAAAACA
AAAGTAAGAAAAAGGCACAGCAAGCAGCAGCTGACACAGGAAACAACAGCCAGGTCAGCCAAAATTAC
CCTATAGTGCAGAACCTCCAGGGGCAAATGGTACATCAGGCCATATCACCTAGAACTTTAAATGCATGG
GTAAAAGTAGTAGAAGAGAAGGCTTTCAGCCCAGAAGTAATACCCATGTTTTCAGCATTATCAGAAGGA
GCCACCCCACAAGATTTAAATACCATGCTAAACACAGTGGGGGGACATCAAGCAGCCATGCAAATGTTA
AAAGAGACCATCAATGAGGAAGCTGCAGAATGGGATAGATTGCATCCAGTGCATGCAGGGCCTATTGC
ACCAGGCCAGATGAGAGAACCAAGGGGAAGTGACATAACAGGAACTACTAGTACCCTTCAGGAACAAA
TAGGATGGATGACACATAATCCACCTATCCCAGTAGGAGAAATCTATAAAAGATGGATAATCCTGGGAT
TAAATAAAATAGTAAGAATGTATAGCCCTACCAGCATTCTGGACATAAGACAAGGACCAAAGGAACCCT
TTAGAGACTATGTAGACCGATTCTATAAAACTCTAAGAGCCGAGCAAGCTTCACAAGAGGTAAAAAATT
GGATGACAGAAACCTTGTTGGTCCAAAATGCGAACCCAGATTGTAAGACTATTTTAAAAGCATTGGGAC
CAGGAGCGACACTAGAAGAAATGATGACAGCATGTCAGGGAGTGGGGGGACCCGGCCATAAAGCAAG
AGTTTTGGCTGAAGCAATGAGCCAAGTAACAAATCCAGCTACCATAATGATACAGAAAGGCAATTTTAG
GAACCAAAGAAAGACTGTTAAGTGTTTCAATTGTGGCAAAGAAGGGCACATAGCCAAAAATTGCAGGG
CCCCTAGGAAAAAGGGCTGTTGGAAATGTGGAAAGGAAGGACACCAAATGAAAGATTGTACTGAGAG
ACAGGCTAATTTTTTAGGGAAGATCTGGCCTTCCCACAAGGGAAGGCCAGGGAATTTTCTTCAGAGCAG
ACCAGAGCCAACAGCCCCACCAGAAGAGAGCTTCAGGTTTGGGGAAGAGACAACAACTCCCTCTCAGA
AGCAGGAGCCGATAGACAAGGAACTGTATCCTTTAGCTTCCCTCAGATCACTCTTTGGCAGCGACCCCTC
GTCACAATAAAGATAGGGGGGCAATTAAAGGAAGCTCTATTAGATACAGGAGCAGATGATACAGTATT
AGAAGAAATGAATTTGCCAGGAAGATGGAAACCAAAAATGATAGGGGGAATTGGAGGTTTTATCAAAG
TAAGACAGTATGATCAGATACTCATAGAAATCTGCGGACATAAAGCTATAGGTACAGTATTAGTAGGAC
CTACACCTGTCAACATAATTGGAAGAAATCTGTTGACTCAGATTGGCTGCACTTTAAATTTTCCCATTAGT
CCTATTGAGACTGTACCAGTAAAATTAAAGCCAGGAATGGATGGCCCAAAAGTTAAACAATGGCCATTG
ACAGAAGAAAAAATAAAAGCATTAGTAGAAATTTGTACAGAAATGGAAAAGGAAGGAAAAATTTCAAA
AATTGGGCCTGAAAATCCATACAATACTCCAGTATTTGCCATAAAGAAAAAAGACAGTACTAAATGGAG
AAAATTAGTAGATTTCAGAGAACTTAATAAGAGAACTCAAGATTTCTGGGAAGTTCAATTAGGAATACC
ACATCCTGCAGGGTTAAAACAGAAAAAATCAGTAACAGTACTGGATGTGGGCGATGCATATTTTTCAGT

TCCCTTAGATAAAGACTTCAGGAAGTATACTGCATTTACCATACCTAGTATAAACAATGAGACACCAGGG
ATTAGATATCAGTACAATGTGCTTCCACAGGGATGGAAAGGATCACCAGCAATATTCCAGTGTAGCATG
ACAAAAATCTTAGAGCCTTTTAGAAAACAAAATCCAGACATAGTCATCTATCAATACATGGATGATTTGT
ATGTAGGATCTGACTTAGAAATAGGGCAGCATAGAACAAAAATAGAGGAACTGAGACAACATCTGTTG
AGGTGGGGATTTACCACACCAGACAAAAAACATCAGAAAGAACCTCCATTCCTTTGGATGGGTTATGAA
CTCCATCCTGATAAATGGACAGTACAGCCTATAGTGCTGCCAGAAAAGGACAGCTGGACTGTCAATGAC
ATACAGAAATTAGTGGGAAAATTGAATTGGGCAAGTCAGATTTATGCAGGGATTAAAGTAAGGCAATTA
TGTAAACTTCTTAGGGGAACCAAAGCACTAACAGAAGTAGTACCACTAACAGAAGAAGCAGAGCTAGA
ACTGGCAGAAAACAGGGAGATTCTAAAAGAACCGGTACATGGAGTGTATTATGACCCATCAAAAGACTT
AATAGCAGAAATACAGAAGCAGGGGCAAGGCCAATGGACATATCAAATTTATCAAGAGCCATTTAAAA
ATCTGAAAACAGGAAAGTATGCAAGAATGAAGGGTGCCCACACTAATGATGTGAAACAATTAACAGAG
GCAGTACAAAAAATAGCCACAGAAAGCATAGTAATATGGGGAAAGACTCCTAAATTTAAATTACCCATA
CAAAAGGAAACATGGGAAGCATGGTGGACAGAGTATTGGCAAGCCACCTGGATTCCTGAGTGGGAGTT
TGTCAATACCCCTCCCTTAGTGAAGTTATGGTACCAGTTAGAGAAAGAACCCATAATAGGAGCAGAAAC
TTTCTATGTAGATGGGGCAGCCAATAGGGAAACTAAATTAGGAAAAGCAGGATATGTAACTGACAGAG
GAAGACAAAAAGTTGTCCCCCTAACGGACACAACAAATCAGAAGACTGAGTTACAAGCAATTCATCTAG
CTTTGCAGGATTCGGGATTAGAAGTAAACATAGTGACAGACTCACAATATGCATTGGGAATCATTCAAG
CACAACCAGATAAGAGTGAATCAGAGTTAGTCAGTCAAATAATAGAGCAGTTAATAAAAAAGGAAAAA
GTCTACCTGGCATGGGTACCAGCACACAAAGGAATTGGAGGAAATGAACAAGTAGATAAATTGGTCAG
TGCTGGAATCAGGAAAGTACTATTTTTAGATGGAATAGATAAGGCCCAAGAAGAACATGAGAAATATCA
CAGTAATTGGAGAGCAATGGCTAGTGATTTTAACCTACCACCTGTAGTAGCAAAAGAAATAGTAGCCAG
CTGTGATAAATGTCAGCTAAAAGGGGAAGCCATGCATGGACAAGTAGACTGTAGCCCAGGAATATGGC
AGCTAGATTGTACACATTTAGAAGGAAAAGTTATCTTGGTAGCAGTTCATGTAGCCAGTGGATATATAG
AAGCAGAAGTAATTCCAGCAGAGACAGGGCAAGAAACAGCATACTTCCTCTTAAAATTAGCAGGAAGA
TGGCCAGTAAAAACAGTACATACAGACAATGGCAGCAATTTCACCAGTACTACAGTTAAGGCCGCCTGT
TGGTGGGCGGGGATCAAGCAGGAATTTGGCATTCCCTACAATCCCCAAAGTCAAGGAGTAATAGAATCT
ATGAATAAAGAATTAAAGAAAATTATAGGACAGGTAAGAGATCAGGCTGAACATCTTAAGACAGCAGT
ACAAATGGCAGTATTCATCCACAATTTTAAAAGAAAAGGGGGGATTGGGGGGTACAGTGCAGGGGAAA
GAATAGTAGACATAATAGCAACAGACATACAAACTAAAGAATTACAAAAACAAATTACAAAAATTCAAA
ATTTTCGGGTTTATTACAGGGACAGCAGAGATCCAGTTTGGAAAGGACCAGCAAAGCTCCTCTGGAAAG
GTGAAGGGGCAGTAGTAATACAAGATAATAGTGACATAAAAGTAGTGCCAAGAAGAAAAGCAAAGATC
ATCAGGGATTATGGAAAACAGATGGCAGGTGATGATTGTGTGGCAAGTAGACAGGATGAGGATTAACA
CATGGAAAAGATTAGTAAAACACCATATGTATATTTCAAGGAAAGCTAAGGACTGGTTTTATAGACATC
ACTATGAAAATACTAATCCAAAAATAAGTTCAGAAGTACACATCCCACTAGGGGATGCTAAATTAGTAAT
AACAACATATTGGGGTCTGCATACAGGAGAAAGAGACTGGCATTTGGGTCAGGGAGTCTCCATAGAAT
GGAGGAAAAAGAGATATAGCACACAAGTAGACCCTGACCTAGCAGACCAACTAATTCATCTGCACTATT
TTGATTGTTTTTCAGAATCTGCTATAAGAAATACCATATTAGGACGTATAGTTAGTCCTAGGTGTGAATAT
CAAGCAGGACATAACAAGGTAGGATCTCTACAGTACTTGGCACTAGCAGCATTAATAAAACCAAAACAG
ATAAAGCCACCTTTGCCTAGTGTTAGGAAACTGACAGAGGACAGATGGAACAAGCCCCAGAAGACCAA
GGGCCACAGAGGGAGCCATACAATGAATGGACACTAGAGCTTTTAGAGGAACTTAAGAGTGAAGCTGT
TAGACATTTTCCTAGGATATGGCTCCATAACTTAGGACAACATATCTATGAAACTTACGGGGATACTTGG
GCAGGAGTGAAGCCATAATAAGAATTCTGCAACAACTGCTGTTTATCCATTTCAGAATTGGGTGTCGAC
ATAGCAGAATAGGCGTTACTCGACAGAGGAGAGCAAGAAATGGAGCCAGTAGATCCTAGACTAGAGCC
CTGGAAGCATCCAGGAAGTCAGCCTAAAACTGCTTGTACCAATTGCTATTGTAAAAAGTGTTGTTTTCAT

TGCCAAGTTTGTTTTATGACAAAAGCCTTAGGCATCTCCTATGGCAGGAAGAAGCGGAGACAGCGACGA
AGAGCTCATCAGAACAGTCAGACTCATCAAGCTTCTCTATCAAAGCAGTAAGTAGTACATGTAATGCAAC
CTATAATAGTAGCAATAGTAGCATTAGTAGTAGCAATAATAATAGCAATAGTTGTGTGGTCCATAGTAAT
CATAGAATATAGGAAAATATTAAGACAAAGAAAAATAGACAGGTTAATTGATAGACTAATAGAAAGAG
CAGAAGACAGTGGCAATGAGAGTGAAGGAGAAGTATCAGCACTTGTGGAGATGGGGGTGGAAATGGG
GCACCATGCTCCTTGGGATATTGATGATCTGTAGTGCTACAGAAAAATTGTGGGTCACAGTCTATTATGG
GGTACCTGTGTGGAAGGAAGCAACCACCACTCTATTTTGTGCATCAGATGCTAAAGCATATGATACAGA
GGTACATAATGTTTGGGCCACACATGCCTGTGTACCCACAGACCCCAACCCACAAGAAGTAGTATTGGT
AAATGTGACAGAAAATTTTAACATGTGGAAAAATGACATGGTAGAACAGATGCATGAGGATATAATCA
GTTTATGGGATCAAAGCCTAAAGCCATGTGTAAAATTAACCCCACTCTGTGTTAGTTTAAAGTGCACTGA
TTTGAAGAATGATACTAATACCAATAGTAGTAGCGGGAGAATGATAATGGAGAAAGGAGAGATAAAAA
ACTGCTCTTTCAATATCAGCACAAGCATAAGACATAAGGTGCAGAAAGAATATGCATTCTTTTATAAACT
TGATATAGTACCAATAGATAATACCAGCTATAGGTTGATAAGTTGTAACACCTCAGTCATTACACAGGCC
TGTCCAAAGGTATCCTTTGAGCCAATTCCCATACATTATTGTGCCCCGGCTGGTTTTGCGATTCTAAAATG
TAATAATAAGACGTTCAATGGAACAGGACCATGTACAAATGTCAGCACAGTACAATGTACACATGGAAT
CAGGCCAGTAGTATCAACTCAACTGCTGTTAAATGGCAGTCTAGCAGAAGAAGATGTAGTAATTAGATC
TGCCAATTTCACAGACAATGCTAAAACCATAATAGTACAGCTGAACACATCTGTAGAAATTAATTGTACA
AGACCCAACAACAATACAAGAAAAAGTATCCGTATCCAGAGGGGACCAGGGAGAGCATTTGTTACAAT
AGGAAAAATAGGAAATATGAGACAAGCACATTGTAACATTAGTAGAGCAAAATGGAATGCCACTTTAA
AACAGATAGCTAGCAAATTAAGAGAACAATTTGGAAATAATAAAACAATAATCTTTAAGCAATCCTCAG
GAGGGGACCCAGAAATTGTAACGCACAGTTTTAATTGTGGAGGGGAATTTTTCTACTGTAATTCAACAC
AACTGTTTAATAGTACTTGGTTTAATAGTACTTGGAGTACTGAAGGGTCAAATAACACTGAAGGAAGTG
ACACAATCACACTCCCATGCAGAATAAAACAATTTATAAACATGTGGCAGGAAGTAGGAAAAGCAATGT
ATGCCCCTCCCATCAGTGGACAAATTAGATGTTCATCAAATATTACTGGGCTGCTATTAACAAGAGATGG
TGGTAATAACAACAATGGGTCCGAGATCTTCAGACCTGGAGGAGGCGATATGAGGGACAATTGGAGAA
GTGAATTATATAAATATAAAGTAGTAAAAATTGAACCATTAGGAGTAGCACCCACCAAGGCAAAGAGAA
GAGTGGTGCAGAGAGAAAAAAGAGCAGTGGGAATAGGAGCTTTGTTCCTTGGGTTCTTGGGAGCAGCA
GGAAGCACTATGGGCGCAGCGTCAATGACGCTGACGGTACAGGCCAGACAATTATTGTCTGATATAGT
GCAGCAGCAGAACAATTTGCTGAGGGCTATTGAGGCGCAACAGCATCTGTTGCAACTCACAGTCTGGG
GCATCAAACAGCTCCAGGCAAGAATCCTGGCTGTGGAAAGATACCTAAAGGATCAACAGCTCCTGGGG
ATTTGGGGTTGCTCTGGAAAACTCATTTGCACCACTGCTGTGCCTTGGAATGCTAGTTGGAGTAATAAAT
CTCTGGAACAGATTTGGAATAACATGACCTGGATGGAGTGGGACAGAGAAATTAACAATTACACAAGCT
TAATACACTCCTTAATTGAAGAATCGCAAAACCAGCAAGAAAAGAATGAACAAGAATTATTGGAATTAG
ATAAATGGGCAAGTTTGTGGAATTGGTTTAACATAACAAATTGGCTGTGGTATATAAAATTATTCATAAT
GATAGTAGGAGGCTTGGTAGGTTTAAGAATAGTTTTTGCTGTACTTTCTATAGTGAATAGAGTTAGGCA
GGGATATTCACCATTATCGTTTCAGACCCACCTCCCAATCCCGAGGGGACCCGACAGGCCCGAAGGAAT
AGAAGAAGAAGGTGGAGAGAGAGACAGAGACAGATCCATTCGATTAGTGAACGGATCCTTAGCACTTA
TCTGGGACGATCTGCGGAGCCTGTGCCTCTTCAGCTACCACCGCTTGAGAGACTTACTCTTGATTGTAAC
GAGGATTGTGGAACTTCTGGGACGCAGGGGGTGGGAAGCCCTCAAATATTGGTGGAATCTCCTACAGT
ATTGGAGTCAGGAACTAAAGAATAGTGCTGTTAACTTGCTCAATGCCACAGCCATAGCAGTAGCTGAGG
GGACAGATAGGGTTATAGAAGTATTACAAGCAGCTTATAGAGCTATTCGCCACATACCTAGAAGAATAA
GACAGGGCTTGGAAAGGATTTTGCTATAAGATGGGTGGCAAGTGGTCAAAAAGTAGTGTGATTGGATG
GCCTGCTGTAAGGGAAAGAATGAGACGAGCTGAGCCAGCAGCAGATGGGGTGGGAGCAGTATCTCGA
GACCTAGAAAAACATGGAGCAATCACAAGTAGCAATACAGCAGCTAACAATGCTGCTTGTGCCTGGCTA

GAAGCACAAGAGGAGGAAGAGGTGGGTTTTCCAGTCACACCTCAGGTACCTTTAAGACCAATGACTTAC
AAGGCAGCTGTAGATCTTAGCCACTTTTTAAAAGAAAAGGGGGGACTGGAAGGGCTAATTCACTCCCAA
AGAAGACAAGATATCCTTGATCTGTGGATCTACCACACACAAGGCTACTTCCCTGATTGGCAGAACTACA
CACCAGGGCCAGGGGTCAGATATCCACTGACCTTTGGATGGTGCTACAAGCTAGTACCAGTTGAGCCAG
ATAAGGTAGAAGAGGCCAATAAAGGAGAGAACACCAGATTGTTACACCCTGTGAGCCTGCATGGAATG
GATGACCCTGAGAGAGAAGTGTTAGAGTGGAGGTTTGACAGCCGCCTAGCATTTCATCACGTGGCCCG
AGAGCTGCATCCGGAGTACTTCAAGAACTGCTGACATCGAGCTTGCTACAAGGGACTTTCCGCTGGGGA
CTTTCCAGGGAGGCGTGGCCTGGTGCTACAAGGAACTTTCCGCTGGGGACTTTCCAGGGAGGCGTGGC
CTGGGCGGGACTGGGGAGTGGCGAGCCCTCAGATGCTGTATATAAGCAGCTGCTTTTTGCCTGTACGG
GGTCTCTCTGGTTAGACCAGATCTGAGCCTGGGAGCTCTCTGGCTAACTAGGGAACCCACTGCTTAAGC
CTCAATAAAGCTTGCCTTGAGTGCTTCAAGTAGTGTGTGCCCGTCTGTTGTGTGACTCTGGTAACTAGAG
ATCCCTCAGACCCTTTTAGTCAGTGTGGAAAATCTCTAGCA

MC2

TGGAAGGGCTAATTTGGTCCCAAAAAAGACAAGAGATCCTTGATCTGTGGATCTACCACACACAAGGCT
ACTTCCCTGATTGGCAGAACTACACACCAGGGCCAGGGATCAGATATCCACTGACCTTTGGATGGTGCTT
CAAGTTAGTACCAGTTGAACCAGAGCAAGTAGAAGAGGCCAATGAAGGAGAGAACAACAGATTGTTAC
ACCCTATGAGCCAGCATGGGATGGAGGACCCGGAGGGAGAAGTATTAGTGTGGAAGTTTAACAGCCTC
CTAGCATTTCGTCACATGGCCCGAGAGCTGCATCCGGAGTACGACAAAGACTGCTGACATCGAGCTTTC
TACAAGGGACTTTCCGCTGGGGACTTTCCAGGGAGGTGTGGCCTGGGCGGGACTGGGGAGTGGCGAG
CCCTCAGATACTATATATAAGCAGCTGCTTTTTGCCTGTACGGGGTCTCTCTGGTTAGACCAGATCTGAG
CCTGGGAGCTCTCTGGCTAACTAGGGAACCCACTGCTTAAGCCTCAATAAAGCTTGCCTTGAGTGCTCAA
AGTAGTGTGTGCCCGTCTGTTGTGTGACTCTGGTAACTAGAGATCCCTCAGACCCTTTTAGTCAGTGTGG
AAAATCTCTAGCAGTGGCGCCCGAACAGGGACTTGAAAGCGAAAGTAAAGCCAGAGGAGATCTCTCGA
CGCAGGACTCGGCTTGCTGAAGCGCGCACGGCAAGAGGCGAGGGGCGGCGACTGGTGAGTACGCCAA
AAATTTTGACTAGCGGAGGCTAGAAGGAGAGAGATGGGTGCGAGAGCGTCGGTATTAAGCGGGGGAG
AATTAGATAAATGGGAAAAAATTCGGTTAAGGCCAGGGGGAAAGAAACAATATAAACTAAAACATATA
GTATGGGCAAGCAGGGAGCTAGAACGATTCGCAGTTAATCCTGGCCTTTTAGAGACATCAGAAGGCTGT
AGACAAATACTGGGACAGCTACAACCATCCCTTCAGACAGGATCAGAAGAACTTAGATCATTATATAAT
ACAATAGCAGTCCTCTATTGTGTGCATCAAAGGATAGATGTAAAAGACACCAAGGAAGCCTTAGATAAG
ATAGAGGAAGAGCAAAACAAAAGTAAGAAAAAGGCACAGCAAGCAGCAGCTGACACAGGAAACAACA
GCCAGGTCAGCCAAAATTACCCTATAGTGCAGAACCTCCAGGGGCAAATGGTACATCAGGCCATATCAC
CTAGAACTTTAAATGCATGGGTAAAAGTAGTAGAAGAGAAGGCTTTCAGCCCAGAAGTAATACCCATGT
TTTCAGCATTATCAGAAGGAGCCACCCCACAAGATTTAAATACCATGCTAAACACAGTGGGGGGACATC
AAGCAGCCATGCAAATGTTAAAAGAGACCATCAATGAGGAAGCTGCAGAATGGGATAGATTGCATCCA
GTGCATGCAGGGCCTATTGCACCAGGCCAGATGAGAGAACCAAGGGGAAGTGACATAACAGGAACTAC
TAGTACCCTTCAGGAACAAATAGGGTGGATGACACATAATCCACCTATCCCAGTAGGAGAAATCTATAA
AAGATGGATAATCCTGGGATTAAATAAAATAGTAAGAATGTATAGCCCTACCAGCATTCTGGACATAAG
ACAAGGACCAAAGGAACCCTTTAGAGACTATGTAGACCGATTCTATAAAACTCTAAGAGCCGAGCAAGC
TTCACAAGAGGTAAAAAATTGGATGACAGAAACCTTGTTGGTCCAAAATGCGAACCCAGATTGTAAGAC
TATTTTAAAAGCATTGGGACCAGGAGCGACACTAGAAGAAATGATGACAGCATGTCAGGGAGTGGGGG
GACCCGGCCATAAAGCAAGAGTTTTGGCTGAAGCAATGAGCCAAGTAACAAATCCAGCTACCATAATGA
TACAGAAAGGCAATTTTAGGAACCAAAGAAAGACTGTTAAGTGTTTCAATTGTGGCAAAGAAGGGCAC

ATAGCCAAAAATTGCAGGGCCCCTAGGAAAAAGGGCTGTTGGAAATGTGGAAAGGAAGGACACCAAAT
GAAAGATTGTACTGAGAGACAGGCTAATTTTTTAGGGAAGATCTGGCCTTCCCACAAGGGAAGGCCAG
GGAATTTTCTTCAGAGCAGACCAGAGCCAACAGCCCCACCAGAAGAGAGCTTCAGGTTTGGGGAAGAG
ACAACAACTCCCTCTCAGAAGCAGGAGCCGATAGACAAGGAACTGTATCCTTTAGCTTCCCTCAGATCAC
TCTTTGGCAGCGACCCCTCGTCACAATAAAGATAGGGGGGCAATTAAAGGAAGCTCTATTAGATACAGG
AGCAGATGATACAGTATTAGAAGAAATGAATTTGCCAGGAAGATGGAAACCAAAAATGATAGGGGGAA
TTGGAGGTTTTATCAAAGTAAGACAGTATGATCAGATACTCATAGAAATCTGCGGACATAAAGCTATAG
GTACAGTATTAGTAGGACCTACACCTGTCAACATAATTGGAAGAAATCTGTTGACTCAGATTGGCTGCAC
TTTAAATTTTCCCATTAGTCCTATTGAGACTGTACCAGTAAAATTAAAGCCAGGAATGGATGGCCCAAAA
GTTAAACAATGGCCATTGACAGAAGAAAAAATAAAAGCATTAGTAGAAATTTGTACAGAAATGGAAAA
GGAAGGAAAAATTTCAAAAATTGGGCCTGAAAATCCATACAATACTCCAGTATTTGCCATAAAGAAAAA
AGACAGTACTAAATGGAGAAAATTAGTAGATTTCAGAGAACTTAATAAGAGAACTCAAGATTTCTGGGA
AGTTCAATTAGGAATACCACATCCTGCAGGGTTAAAACAGAAAAAATCAGTAACAGTACTGGATGTGGG
CGATGCATATTTTTCAGTTCCCTTAGATAAAGACTTCAGGAAGTATACTGCATTTACCATACCTAGTATAA
ACAATGAGACACCAGGGATTAGATATCAGTACAATGTGCTTCCACAGGGATGGAAAGGATCACCAGCA
ATATTCCAGTGTAGCATGACAAAAATCTTAGAGCCTTTTAGAAAACAAAATCCAGACATAGTCATCTATC
AATACATGGATGATTTGTATGTAGGATCTGACTTAGAAATAGGGCAGCATAGAACAAAAATAGAGGAA
CTGAAACAACATCTGTTGAGGTGGGGATTTACCACACCAGACAAAAAACATCAGAAAGAACCTCCATTC
CTTTGGATGGGTTATGAACTCCATCCTGATAAATGGACAGTACAGCCTATAGTGCTGCCAGAAAAGGAC
AGCTGGACTGTCAATGACATACAGAAATTAGTGGGAAAATTGAATTGGGCAAGTCAGATTTATGCAGG
GATTAAAGTAAGGCAATTATGTAAACTTCTTAGGGGAACCAAAGCACTAACAGAAGTAGTACCACTAAC
AGAAGAAGCAGAGCTAGAACTGGCAGAAAACAGGGAGATTCTAAAAGAACCGGTACATGGAGTGTATT
ATGACCCATCAAAAGACTTAATAGCAGAAATACAGAAGCAGGGGCAAGGCCAATGGACATATCAAATTT
ATCAAGAGCCATTTAAAAATCTGAAAACAGGAAAGTATGCAAGAATGAAGGGTGCCCACACTAATGAT
GTGAAACAATTAACAGAGGCAGTACAAAAAATAGCCACAGAAAGCATAGTAATATGGGGAAAGACTCC
TAAATTTAAATTACCCATACAAAAGGAAACATGGGAAGCATGGTGGACAGAGTATTGGCAAGCCACCTG
GATTCCTGAGTGGGAGTTTGTCAATACCCCTCCCTTAGTGAAGTTATGGTACCAGTTAGAGAAAGAACCC
ATAATAGGAGCAGAAACTTTCTATGTAGATGGGGCAGCCAATAGGGAAACTAAATTAGGAAAAGCAGG
ATATGTAACTGACAGAGGAAGACAAAAAGTTGTCCCCCTAACGGACACAACAAATCAGAAGACTGAGTT
ACAAGCAATTCATCTAGCTTTGCAGGATTCGGGATTAGAAGTAAACATAGTGACAGACTCACAATATGC
ATTGGGAATCATTCAAGCACAACCAGATAAGAGTGAATCAGAGTTAGTCAGTCAAATAATAGAGCAGTT
AATAAAAAAGGAAAAAGTCTACCTGGCATGGGTACCAGCACACAAAGGAATTGGAGGAAATGAACAAG
TAGATAAATTGGTCAGTGCTGGAATCAGGAAAGTACTATTTTTAGATGGAATAGATAAGGCCCAAGAAG
AACATGAGAAATATCACAGTAATTGGAGAGCAATGGCTAGTGATTTTAACCTACCACCTGTAGTAGCAA
AAGAAATAGTAGCCAGCTGTGATAAATGTCAGCTAAAAGGGGAAGCCATGCATGGACAAGTAGACTGT
AGCCCAGGAATATGGCAGCTAGATTGTACACATTTAGAAGGAAAAGTTATCTTGGTAGCAGTTCATGTA
GCCAGTGGATATATAGAAGCAGAAGTAATTCCAGCAGAGACAGGGCAAGAAACAGCATACTTCCTCTTA
AAATTAGCAGGAAGATGGCCAGTAAAAACAGTACATACAGACAATGGCAGCAATTTCACCAGTACTACA
GTTAAGGCCGCCTGTTGGTGGGCGGGGATCAAGCAGGAATTTGGCATTCCCTACAATCCCCAAAGTCAA
GGAGTAATAGAATCTATGAATAAAGAATTAAAGAAAATTATAGGACAGGTAAGAGATCAGGCTGAACA
TCTTAAGACAGCAGTACAAATGGCAGTATTCATCCACAATTTTAAAAGAAAAGGGGGGATTGGGGGGT
ACAGTGCAGGGGAAAGAATAGTAGACATAATAGCAACAGACATACAAACTAAAGAATTACAAAAACAA
ATTACAAAAATTCAAAATTTTCGGGTTTATTACAGGGACAGCAGAGATCCAGTTTGGAAAGGACCAGCA
AAGCTCCTCTGGAAAGGTGAAGGGGCAGTAGTAATACAAGATAATAGTGACATAAAAGTAGTGCCAAG

AAGAAAAGCAAAGATCATCAGGGATTATGGAAAACAGATGGCAGGTGATGATTGTGTGGCAAGTAGAC
AGGATGAGGATTAACACATGGAAAAGATTAGTAAAACACCATATGTATATTTCAAGGAAAGCTAAGGAC
TGGTTTTATAGACATCACTATGAAAATACTAATCCAAAAATAAGTTCAGAAGTACACATCCCACTAGGGG
ATGCTAAATTAGTAATAACAACATATTGGGGTCTGCATACAGGAGAAAGAGACTGGCATTTGGGTCAGG
GAGTCTCCATAGAATGGAGGAAAAAGAGATATAGCACACAAGTAGACCCTGACCTAGCAGACCAACTA
ATTCATCTGCACTATTTTGATTGTTTTTCAGAATCTGCTATAAGAAATACCATATTAGGACGTATAGTTAG
TCCTAGGTGTGAATATCAAGCAGGACATAACAAGGTAGGATCTCTACAGTACTTGGCACTAGCAGCATT
AATAAAACCAAAACAGATAAAGCCACCTTTGCCTAGTGTTAGGAAACTGACAGAGGACAGATGGAACA
AGCCCCAGAAGACCAAGGGCCACAGAGGGAGCCATACAATGAATGGACACTAGAGCTTTTAGAGGAAC
TTAAGAGTGAAGCTGTTAGACATTTTCCTAGGATATGGCTCCATAACTTAGGACAACATATCTATGAAAC
TTACGGGGATACTTGGGCAGGAGTGAAGCCATAATAAGAATTCTGCAACAACTGCTGTTTATCCATTTCA
GAATTGGGTGTCGACATAGCAGAATAGGCGTTACTCGACAGAGGAGAGCAAGAAATGGAGCCAGTAG
ATCCTAGACTAGAGCCCTGGAAGCATCCAGGAAGTCAGCCTAAAACTGCTTGTACCAATTGCTATTGTAA
AAAGTGTTGCTTTCATTGCCAAGTTTGTTTTATGACAAAAGCCTTAGGCATCTCCTATGGCAGGAAGAAG
CGGAGACAGCGACGAAGAGCTCATCAGAACAGTCAGACTCATCAAGCTTCTCTATCAAAGCAGTAAGTA
GTACATGTAATGCAACCTATAATAGTAGCAATAGTAGCATTAGTAGTAGCAATAATAATAGCAATAGTTG
TGTGGTCCATAGTAATCATAGAATATAGGAAAATATTAAGACAAAGAAAAATAGACAGGTTAATTGATA
GACTAATAGAAAGAGCAGAAGACAGTGGCAATGAGAGTGAAGGAGAAGTATCAGCACTTGTGGAGAT
GGGGGTGGAAATGGGGCACCATGCTCCTTGGGATATTGATGATCTGTAGTGCTACAGAAAAATTGTGG
GTCACAGTCTATTATGGGGTACCTGTGTGGAAGGAAGCAACCACCACTCTATTTTGTGCATCAGATGCTA
AAGCATATGATACAGAGGTACATAATGTTTGGGCCACACATGCCTGTGTACCCACAGACCCCAACCCAC
AAGAAGTAGTATTGGTAAATGTGACAGAAAATTTTAACATGTGGAAAAATGACATGGTAGAACAGATG
CATGAGGATATAATCAGTTTATGGGATCAAAGCCTAAAGCCATGTGTAAAATTAACCCCACTCTGTGTTA
GTTTAAAGTGCACTGATTTGAAGAATGATACTAATACCAATAGTAGTAGCGGGAGAATGATAATGGAGA
AAGGAGAGATAAAAAACTGCTCTTTCAATATCAGCACAAGCATAAGACATAAGGTGCAGAAAGAATAT
GCATTCTTTTATAAACTTGATATAGTACCAATAGATAATACCAGCTATAGGTTGATAAGTTGTAACACCTC
AGTCATTACACAGGCCTGTCCAAAGGTATCCTTTGAGCCAATTCCCATACATTATTGTGCCCCGGCTGGTT
TTGCGATTCTAAAATGTAATAATAAGACGTTCAATGGAACAGGACCATGTACAAATGTCAGCACAGTAC
AATGTACACATGGAATCAGGCCAGTAGTATCAACTCAACTGCTGTTAAATGGCAGTCTAGCAGAAGAAG
ATGTAGTAATTAGATCTGCCAATTTCACAGACAATGCTAAAACCATAATAGTACAGCTGAACACATCTGT
AGAAATTAATTGTACAAGACCCAACAACAATACAAGAAAAAGTATCCGTATCCAGAGGGGACCAGGGA
GAGCATTTGTTACAATAGGAAAAATAGGAAATATGAGACAAGCACATTGTAACATTAGTAGAGCAAAAT
GGAATGCCACTTTAAAACAGATAGCTAGCAAATTAAGAGAACAATTTGGAAATAATAAAACAATAATCT
TTAAGCAATCCTCAGGAGGGGACCCAGAAATTGTAACGCACAGTTTTAATTGTGGAGGGGAATTTTTCT
ACTGTAATTCAACACAACTGTTTAATAGTACTTGGTTTAATAGTACTTGGAGTACTGAAGGGTCAAATAA
CACTGAAGGAAGTGACACAATCACACTCCCATGCAGAATAAAACAATTTATAAACATGTGGCAGGAAGT
AGGAAAAGCAATGTATGCCCCTCCCATCAGTGGACAAATTAGATGTTCATCAAATATTACTGGGCTGCTA
TTAACAAGAGATGGTGGTAATAACAACAATGGGTCCGAGATCTTCAGACCTGGAGGAGGCGATATGAG
GGACAATTGGAGAAGTGAATTATATAAATATAAAGTAGTAAAAATTGAACCATTAGGAGTAGCACCCAC
CAAGGCAAAGAGAAGAGTGGTGCAGAGAGAAAAAAGAGCAGTGGGAATAGGAGCTTTGTTCCTTGGG
TTCTTGGGAGCAGCAGGAAGCACTATGGGCGCAGCGTCAATGACGCTGACGGTACAGGCCAGACAATT
ATTGTCTGATATAGTGCAGCAGCAGAACAATTTGCTGAGGGCTATTGAGGCGCAACAGCATCTGTTGCA
ACTCACAGTCTGGGGCATCAAACAGCTCCAGGCAAGAATCCTGGCTGTGGAAAGATACCTAAAGGATCA
ACAGCTCCTGGGGATTTGGGGTTGCTCTGGAAAACTCATTTGCACCACTGCTGTGCCTTGGAATGCTAGT

TGGAGTAATAAATCTCTGGAACAGATTTGGAATAACATGACCTGGATGGAGTGGGACAGAGAAATTAA
CAATTACACAAGCTTAATACACTCCTTAATTGAAGAATCGCAAAACCAGCAAGAAAAGAATGAACAAGA
ATTATTGGAATTAGATAAATGGGCAAGTTTGTGGAATTGGTTTAACATAACAAATTGGCTGTGGTATATA
AAATTATTCATAATGATAGTAGGAGGCTTGGTAGGTTTAAGAATAGTTTTTGCTGTACTTTCTATAGTGA
ATAGAGTTAGGCAGGGATATTCACCATTATCGTTTCAGACCCACCTCCCAATCCCGAGGGGACCCGACA
GGCCCGAAGGAATAGAAGAAGAAGGTGGAGAGAGAGACAGAGACAGATCCATTCGATTAGTGAACGG
ATCCTTAGCACTTATCTGGGACGATCTGCGGAGCCTGTGCCTCTTCAGCTACCACCGCTTGAGAGACTTA
CTCTTGATTGTAACGAGGATTGTGGAACTTCTGGGACGCAGGGGGTGGGAAGCCCTCAAATATTGGTG
GAATCTCCTACAGTATTGGAGTCAGGAACTAAAGAATAGTGCTGTTAACTTGCTCAATGCCACAGCCATA
GCAGTAGCTGAGGGGACAGATAGGGTTATAGAAGTATTACAAGCAGCTTATAGAGCTATTCGCCACATA
CCTAGAAGAATAAGACAGGGCTTGGAAAGGATTTTGCTATAAGATGGGTGGCAAGTGGTCAAAAAGTA
GTGTGATTGGATGGCCTGCTGTAAGGGAAAGAATGAGACGAGCTGAGCCAGCAGCAGATGGGGTGGG
AGCAGTATCTCGAGACCTAGAAAAACATGGAGCAATCACAAGTAGCAATACAGCAGCTAACAATGCTGC
TTGTGCCTGGCTAGAAGCACAAGAGGAGGAAGAGGTGGGTTTTCCAGTCACACCTCAGGTACCTTTAAG
ACCAATGACTTACAAGGCAGCTGTAGATCTTAGCCACTTTTTAAAAGAAAAGGGGGGACTGGAAGGGC
TAATTCACTCCCAAAGAAGACAAGATATCCTTGATCTGTGGATCTACCACACACAAGGCTACTTCCCTGA
TTGGCAGAACTACACACCAGGGCCAGGGGTCAGATATCCACTGACCTTTGGATGGTGCTACAAGCTAGT
ACCAGTTGAGCCAGATAAGGTAGAAGAGGCCAATAAAGGAGAGAACACCAGATTGTTACACCCTGTGA
GCCTGCATGGAATGGATGACCCTGAGAGAGAAGTGTTAGAGTGGAGGTTTAACAGCCGCCTAGCATTT
CATCACGTGGCCCGAGAGCTGCATCCGGAGTACGTCAAGAACTGCTGACATCGAGCTTGCTACAAGGG
ACTTTCCGCTGGGGACTTTCCAGGGAGGCGTGGCCTGGGCGGGACTGGGGAGTGGCGAGCCCTCAGAT
ACTGTATATAAGCAGCTGCTTTTTGCCTGTACGGGTCTCTCTGGTTAGACCAGATCTGAGCCTGGGAGC
TCTCTGGCTAACTAGGGAACCCACTGCTTAAGCCTCAATAAAGCTTGCCTTGAGTGCTTCAAGTAGTGTG
TGCCCGTCTGTTGTGTGACTCTGGTAACTAGAGATCCCTCAGACCCTTTTAGTCAGTGTGGAAAATCTCT
AGCA
