# Supplementary material for: Resistance to the Tat Inhibitor Didehydro-Cortistatin A Is Mediated by Heightened Basal HIV-1 Transcription
Source: mBio. 2019 Jul 2;10(4):e01750-18. doi: 10.1128/mBio.01750-18 (PMC6606815; doi:10.1128/mBio.01750-18)
Supplement: TABLE S1 [file mBio.01750-18-st001.pdf]

Table S1. Cloning strategy. Each donor vector was digested with the indicated restriction enzymes and the released fragment containing the insert was cloned into the acceptor vector. Nomenclature based on pNL4-3 numbering.

Final Vector	Donor vector (Insert)	Restriction enzymes	Cloned in acceptor vector	Description
**pNL4-3 2A**	pET28b-NL4-3-MUT2	BssHII/AgeI-HF	pNL4-3	**pNL4-3** with GAG G1498A, A1533G, and POL G3166A
**pNL4-3 2B**	pET28b-NL4-3-MUT2	AgeI-HF/NheI	pNL4-3	**pNL4-3** with VIF G5177A, VPR G5730 deletion, TAT C5943T, and ENV G6719C
**pNL4-3 2C**	pET28b-NL4-3-MUT2	NheI-HF/NgoMIV	pNL4-3	**pNL4-3** with NEF/LTR C9275A, G9342A, T9393G, and LTR G9496A, C9500T, T9528G
**pNL4-3 2D**	pET28b-NL4-3-MUT2	AgeI/NheI	pNL4-3 2A	**pNL4-3 2B** with GAG G1498A, A1533G, and POL G3166A
**pNL4-3 2E**	pET28b-NL4-3-MUT2	NheI/NgoMIV	pNL4-3 2B	**pNL4-3 2B** with NEF/LTR C9275A, G9342A, T9393G, and LTR G9496A, C9500T, T9528G
**pcDNA4 Vif/Vpr mutE**	pNL4-3 2E	SpeI-HF/EcoRI-HF	pcDNA4/TO/myc-hisB	SpeI/EcoRI insert contains NL4-3 sequence with VIF G5177A and VPR G5730 deletion
**pNL4-3 2E Tat/Env**	pNL4-3	EcoRI-HF/NheI-HF	pNL4-3 2E	**pNL4-3 2E** with WT TAT C5943 and WT ENV G6719
**pNL4-3 2E5**	pNL4-3	EcoRI-HF/NheI-HF	pNL4-3 2E Tat/Env	**pNL4-3 2E** with WT VIF G5177, WT TAT C5945 and WT ENV G6719.
**pNL4-3 2E6**	pNL4-3	AgeI-HF/EcoRI-HF	pNL4-3 2E4	**pNL4-3 2E** with WT VIF G5177, WT VPR G5730 and WT ENV G6719.
**pNL4-3 2E7**	pNL4-3	AgeI-HF/EcoRI-HF	pNL4-3 2E3	**pNL4-3 2E** with WT VIF G5177, WT VPR G5730 and WT TAT C5945.
**pNL4-3 2E8**	pNL4-3 2E7	EcoRI-HF/NheI-HF	pNL4-3 2E5	**pNL4-3 2E5** with ENV G6719C
