# Supplementary material for: Resistance to the Tat Inhibitor Didehydro-Cortistatin A Is Mediated by Heightened Basal HIV-1 Transcription
Source: mBio. 2019 Jul 2;10(4):e01750-18. doi: 10.1128/mBio.01750-18 (PMC6606815; doi:10.1128/mBio.01750-18)
Supplement: TABLE S2 [file mBio.01750-18-st002.pdf]

Table S2. Two-steps mutagenesis and cloning strategy. Mutagenesis was performed on the parent plasmid with the indicated primers. The newly mutated plasmid was digested with the indicated restriction enzymes and cloned into the acceptor vector.

**Final vector**	Parent plasmid for mutagenesis	Primer	Primer sequence	Cloned in	Restriction enzymes	Description
**pNL4-3 2E1**	pcDNA4 Vif/Vpr mutE	QC_A5177G_F	actggttttatagacatcactatgaaagtactaatccaaaaataagttcagaag	pNL4-3 2E	AgeI-HF/EcoRI-HF	**pNL4-3 2E** with WT VIF G5177
		QC_A5177G_R	cttctgaacttatttttggattagtactttcatagtgatgtctataaaaccagt			
**pNL4-3 2E2**	pcDNA4 Vif/Vpr mutE	QC_G5730_F	ggatacttgggcaggagtggaagccataataagaattct	pNL4-3 2E	AgeI-HF/EcoRI-HF	**pNL4-3 2E** with WT VPR G5730
		QC_G5730_R	agaattcttattatggcttccactcctgcccaagtatcc			
**pNL4-3 2E3**	pNL4-3 2E Tat/Env	QC_G6719C_F	ttcaatatcagcacaagcataagacataaggtgcagaaagaatatg	pNL4-3 2E	EcoRI-HF/NheI-HF	**pNL4-3 2E** with WT TAT C5943
		QC_G6719C_R	catattctttctgcaccttatgtcttatgcttgtgctgatattgaa			
**pNL4-3 2E4**	pNL4-3 2E Tat/Env	QC_C5943T_F	gctttcattgccaagtttgttttatgacaaaagccttaggcatc	pNL4-3 2E	EcoRI-HF/NheI-HF	**pNL4-3 2E** with WT ENV G6719
		QC_C5943T_R	gatgcctaaggcttttgtcataaaacaaacttggcaatgaaagc			
