# Supplementary material for: Resistance to the Tat Inhibitor Didehydro-Cortistatin A Is Mediated by Heightened Basal HIV-1 Transcription
Source: mBio. 2019 Jul 2;10(4):e01750-18. doi: 10.1128/mBio.01750-18 (PMC6606815; doi:10.1128/mBio.01750-18)
Supplement: TABLE S3 [file mBio.01750-18-st003.pdf]

Table S3. Primers used to analyze the DNA immunoprecipitaed by RNAPII ChIP.

P1-F	CCTTTGGATGGTGCTTCAAGTTAG
P1-R	ATGCTGGCTCATAGGGTGTAAC
P2-F	GGGACTTTCCGCTGGGGAC
P2-R	CCCAGTACAGGCAAAAAGCAGC
P3-F	CCTGTACTGGGTCTCTCTGGTT
P3-R	TTTGAGCACTCAAGGCAAGCTTTA
P4-F	TCTCTGGCTAACTAGGGAACC
P4-R	AAAGGGTCTGAGGGATCTCTAG
P5-F	AGTGTGTGCCCGTCTGTTGTG
P5-R	CTTTCGCTTTCAAGTCCCTGTTCG
P6-F	GCGACTGGTGAGTACGCCAA
P6-R	CCCCTGGCCTTAACCGAATTT
P7-F	CCATCAATGAGGAAGCTGCAGAA
P7-R	GGTGGATTATGTGTCATCCATCCT
P8-F	TTCTTCAGAGCAGACCAGAGC
P8-R	GCTGCCAAAGAGTGATCTGA
P9-F	CAGAAATACAGAAGCAGGGGCAA
P9-R	GTGTGGGCACCCTTCATTCTT
P10-F	TTGCTCAATGCCACAGCCAT
P10-R	TTTGACCACTTGCCACCCAT
RPL13A -F	CATAGGAAGCTGGGAGCAAG
RPL13A - R	GCCCTCCAATCAGTCTTCTG
